# New *Penicillium* and *Talaromyces* species from honey, pollen and nests of stingless bees

**DOI:** 10.1007/s10482-018-1081-1

**Published:** 2018-04-13

**Authors:** Renan N. Barbosa, Jadson D. P. Bezerra, Cristina M. Souza-Motta, Jens C. Frisvad, Robert A. Samson, Neiva T. Oliveira, Jos Houbraken

**Affiliations:** 1 Westerdijk Fungal Biodiversity Institute, Uppsalalaan 8, 3584 CT Utrecht, The Netherlands; 2 Departamento de Micologia Prof. Chaves Batista, Universidade Federal de Pernambuco, Av. Prof. Moraes Rego, s/n, Centro de Biociências, Cidade Universitária, CEP: 50670-901 Recife, PE Brazil; 3 Department of Biotechnology and Biomedicine, Technical University of Denmark, 2800 Kongens Lyngby, Denmark

**Keywords:** 8 new taxa, *Aspergillaceae*, Fungal ecology, Polyphasic approach, Taxonomy, *Trichocomaceae*

## Abstract

*Penicillium* and *Talaromyces* species have a worldwide distribution and are isolated from various materials and hosts, including insects and their substrates. The aim of this study was to characterize the *Penicillium* and *Talaromyces* species obtained during a survey of honey, pollen and the inside of nests of *Melipona scutellaris*. A total of 100 isolates were obtained during the survey and 82% of those strains belonged to *Penicillium* and 18% to *Talaromyces*. Identification of these isolates was performed based on phenotypic characters and β-tubulin and ITS sequencing. Twenty-one species were identified in *Penicillium* and six in *Talaromyces*, including seven new species. These new species were studied in detail using a polyphasic approach combining phenotypic, molecular and extrolite data. The four new *Penicillium* species belong to sections *Sclerotiora* (*Penicillium fernandesiae* sp. nov., *Penicillium mellis* sp. nov., *Penicillium meliponae* sp. nov.) and *Gracilenta* (*Penicillium apimei* sp. nov.) and the three new *Talaromyces* species to sections *Helici* (*Talaromyces pigmentosus* sp. nov.), *Talaromyces* (*Talaromyces mycothecae* sp. nov.) and *Trachyspermi* (*Talaromyces brasiliensis* sp. nov.). The invalidly described species *Penicillium echinulonalgiovense* sp. nov. was also isolated during the survey and this species is validated here.

**Electronic supplementary material:**

The online version of this article (10.1007/s10482-018-1081-1) contains supplementary material, which is available to authorized users.

## Introduction

Stingless bees comprise a diverse group of highly eusocial insects occurring throughout the tropical regions in the world. They are important honey producers and pollinators of several plants (Ramírez et al. 2010; Brown and Oliveira 2014). An example of a stingless bee species is *Melipona scutellaris* (Hymenoptera: Apidae: Meliponini), an indigenous species occurring in the North-eastern part of Brazil and considered to be one of the first species to be domesticated in the Americas (Kerr 1996; Silva et al. 2013). In this part of Brazil, *M. scutellaris* is the main bee species in meliponiculture (stingless beekeeping). Meliponiculture in the rural areas is a sustainable activity and the honey from these bees is widely appreciated as a food source. The composition of the honey of the stingless bees differs from that of bees of the genus *Apis* (honey bees) (Vit et al. 2004). The honey of stingless bees contains, in comparison to honey of honey bees, a more complex mixture of carbohydrates and contains other types of organic acids, proteins, minerals, vitamins, pollen grains and enzymes (Almeida-Muradian et al. 2013). Recently, the interest in honey produced by stingless bees increased. Besides being a food source, also several other functionalities are linked to this type of honey, such as antiseptic, antimicrobial, anti-inflammatory and wound-healing properties (Silva et al. 2013; Rao et al. 2016).

*Penicillium* and *Talaromyces* are fungal genera classified in the order *Eurotiales*. In the dual nomenclature era (pre 2012), *Talaromyces* was known as a sexual genus related to *Penicillium* and other genera. In the last decade, the genera *Talaromyces* and *Penicillium* were re-defined due to new taxonomic insights and the introduction of single name nomenclature (Houbraken and Samson 2011; Samson et al. 2011; McNeill et al. 2012; Yilmaz et al. 2014). Currently, *Penicillium* and *Talaromyces* are separate genera that contain both sexual and asexual species. Visagie et al. (2014) accepted 354 *Penicillium* species and Yilmaz et al. (2014) 88 *Talaromyces* species, and these numbers are rapidly increasing (Houbraken et al. 2016a). Several of the new species that are being discovered are found during ecology and biodiversity studies of specific substrates or habitats (Houbraken et al. 2016a). Describing new species from poorly explored substrates and habitats, like those related to meliponiculture, will add to our knowledge on biodiversity. With this information, future studies will also be able to better understand the ecology of fungi in these type of environments.

Fungi, such as *Penicillium* and *Talaromyces*, can have a strong association to a specific substrate (Peterson et al. 2003; Kobayashi et al. 2008; Visagie 2012, Li et al. 2012; Rivera et al. 2012; Yilmaz et al. 2014). The genera *Aspergillus*, *Penicillium*, *Monascus* and *Mucor* are commonly associated with bees or their products (Egorova 1971; Gilliam et al. 1989; Eltz et al. 2002; Ferraz et al. 2008; Barbosa et al. 2017). Most fungi associated with bees and nests have a saprophytic lifestyle, but fungi can also have a mutualistic relationship with bees (Menezes et al. 2015). On the other hand, fungi are also reported to be pathogenic to many bee species and cause serious problems in honey bee (*Apis mellifera*) brood. *Aspergillus flavus* is the primary species responsible for stonebrood, a disease where dead and mummified larvae are present in the brood cells, but also other Aspergilli such as *Aspergillus fumigatus* and *Aspergillus niger* are reported as aetiological agents of this disease (Gilliam and Vandenberg 1988; Foley et al. 2014; Lopes et al. 2015; Sarwar 2016). Though it is generally accepted that infection only occurs in weakened colonies, the specific conditions predisposing the onset of disease are not fully understood (Shoreit and Bagy 1995).

Fungi play an important role in many ecosystems; however, only a limited number of studies dealt with the association between stingless bees in Brazil and filamentous fungi (e.g. Oliveira and Morato 2000; Ferraz et al. 2008; Góis et al. 2010) and yeasts (e.g. Teixeira et al. 2003; Rosa et al. 2003; Daniel et al. 2013; Barbosa et al. 2016). In the present study, we analysed three different substrates associated with *M. scutellaris* bees: bee pollen, nests and honey. In nature, the *M*. *scutellaris* bee nests are mainly located in tree hollows, and they are kept by beekeepers in artificial wooden hives. The bees use cerumen (a mixture of wax and floral resins) for the construction of their nests and this material is also used inside nests in storage pots, brood cells and entrance openings (Cortopassi-Laurino et al. 2006; Pianaro et al. 2007). The floral pollen is collected, packed into pollen pellets, and subsequently stored inside the nest by worker bees. This stored pollen is referred to as ‘bee bread’. The pollen spectrum has been studied in the past to get insight in the bee colony’s food requirements, pollinating functions and the plant species visited by the bees (Cortopassi-Laurino et al. 2007).

In this paper, we focus on the identification of *Penicillium* and *Talaromyces* species isolated from three different substrates (bee pollen, nests and honey) associated to *M*. *scutellaris* in the Atlantic Rainforest in Brazil. Phenotypic characters, combined with ITS and partial β-tubulin (*BenA*) sequences were applied to identify the isolates. Four *Penicillium* and three *Talaromyces* species could not be assigned to any known species and are described here as new. Those species are described using a polyphasic approach including morphology, ITS, *BenA*, calmodulin (*CaM*) and/or RNA polymerase II second largest subunit (*RPB2*) sequences and extrolites profiles.

## Materials and methods

### Strains

Six collections were performed between January and June 2014 in the tropical forest in Pernambuco, Brazil (8°7′30″S, 34°52′30″W and 8°4′36″S, 34°57′34″W). During each collection, four hives were sampled. Stingless bees process honey and pollen in cerumen pots. Per hive, four samples of the honey pots and four of the pollen pots were collected and combined, resulting in one mixed sample of each substrate. In the same hives, also the surface of brood cells and the pollen and honey pots were sampled using sterile cotton swabs (in total 48 swabs). Analysis of the samples was performed using dichloran 18% glycerol agar (DG18) and malt extract agar supplemented with chloramphenicol as described in Barbosa et al. (2017). The isolates were subsequently deposited in the Micoteca URM culture collection (Federal University of Pernambuco, Recife, Brazil) and ex-type strains in the CBS culture collection, housed at the Westerdijk Fungal Biodiversity Institute, Utrecht, The Netherlands (under Material Transfer Agreement—MTA No. 01/2016/Micoteca URM) (Tables 1, 2). Holotype material (slide preparation) is deposited at Herbário Pe. Camille Torrend (Federal University of Pernambuco, Recife, Brazil). New species names and associated information were deposited in MycoBank.

**Table 1:** Details of strains isolated in this study and used in the phylogenetic analyses

Species	Strain numbers	Substrate; location	Sequence accession numbers			
			ITS	*BenA*	*CaM*	*RPB2*
*Penicillium apimei*	URM 7591 T = CBS 142502	Honey of *Melipona scutellaris*; Recife, Pernambuco, Brazil	MF278310	LT854641	LT882717	LT854650
*Penicillium echinulonalgiovense*	URM 7599	Bee pollen of *Melipona scutellaris*; Recife, Pernambuco, Brazil	MF278311	LT882667	LT882670	LT882673
*Penicillium echinulonalgiovense*	RB 217	Inside nest of *Melipona scutellaris*; Recife, Pernambuco, Brazil	MF278312	LT882668	LT882671	LT882674
*Penicillium echinulonalgiovense*	RB 218	Inside nest of *Melipona scutellaris*; Recife, Pernambuco, Brazil	MF278313	LT882669	LT882672	LT882675
*Penicillium fernandesiae*	URM 7600 T = CBS 142500	Inside nest of *Melipona scutellaris*; Recife, Pernambuco, Brazil	MF278314	LT854645	LT854649	LT854654
*Penicillium meliponae*	URM 7602 T = CBS 142495	Honey of *Melipona scutellaris*; Recife, Pernambuco, Brazil	MF278315	LT854644	LT854648	LT854653
*Penicillium mellis*	URM 7605 T = CBS 142499	Honey of *Melipona scutellaris*; Recife, Pernambuco, Brazil	MF278316	LT854643	LT854647	LT854652
*Penicillium mellis*	URM 7611	Inside nest of *Melipona scutellaris*; Recife, Pernambuco, Brazil	MF278317	LT882629	LT882634	LT882634
*Penicillium mellis*	RB 9	Inside nest of *Melipona scutellaris*; Recife, Pernambuco, Brazil	MF278318	LT882625	LT882630	LT882635
*Penicillium mellis*	RB 69	Honey of *Melipona scutellaris*; Recife, Pernambuco, Brazil	MF278319	LT882626	LT882631	LT882636
*Penicillium mellis*	RB 85	Inside nest of *Melipona scutellaris*; Recife, Pernambuco, Brazil	MF278320	LT882627	LT882632	LT882637
*Penicillium mellis*	RB 110	Inside nest of *Melipona scutellaris*; Recife, Pernambuco, Brazil	MF278321	LT882628	LT882633	LT882638
*Penicillium* sp.	URM 7610 = CBS 142497	Bee pollen of *Melipona scutellaris*; Recife, Pernambuco, Brazil	MF278322	LT882642	LT882646	LT882651
*Talaromyces brasiliensis*	URM 7618 T = CBS 142493	Honey of *Melipona scutellaris*; Recife, Pernambuco, Brazil	MF278323	LT855560	LT855563	LT855566
*Talaromyces brasiliensis*	URM 7619	Inside nest of *Melipona scutellaris*; Recife, Pernambuco, Brazil	MF278324	LT882640	LT882642	LT882644
*Talaromyces brasiliensis*	URM 7620	Inside nest of *Melipona scutellaris*; Recife, Pernambuco, Brazil	MF278325	LT882641	LT882643	LT882645
*Talaromyces mycothecae*	URM 7622 T = CBS 142494	Inside nest of *Melipona scutellaris*; Recife, Pernambuco, Brazil	MF278326	LT855561	LT855564	LT855567
*Talaromyces mycothecae*	URM 7623	Inside nest of *Melipona scutellaris*; Recife, Pernambuco, Brazil	MF278327	LT882646	LT882649	LT882652
*Talaromyces mycothecae*	RB 95	Inside nest of *Melipona scutellaris*; Recife, Pernambuco, Brazil	MF278328	LT882647	LT882650	LT882653
*Talaromyces mycothecae*	RB 171	Inside nest of *Melipona scutellaris*; Recife, Pernambuco, Brazil	MF278329	LT882648	LT882651	LT882654
*Talaromyces pigmentosus*	URM 7624 T = CBS 142805	Inside nest of *Melipona scutellaris*; Recife, Pernambuco, Brazil	MF278330	LT855562	LT855565	LT855568
*Talaromyces pigmentosus*	URM 7625	Bee pollen of *Melipona scutellaris*; Recife, Pernambuco, Brazil	MF278331	LT882655	LT882659	LT882663
*Talaromyces pigmentosus*	RB 30	Inside nest of *Melipona scutellaris*; Recife, Pernambuco, Brazil	MF278332	LT882656	LT882660	LT882664
*Talaromyces pigmentosus*	RB 96	Bee pollen of *Melipona scutellaris*; Recife, Pernambuco, Brazil	MF278333	LT882657	LT882661	LT882665
*Talaromyces pigmentosus*	RB 100	Inside nest of *Melipona scutellaris*; Recife, Pernambuco, Brazil	MF278334	LT882658	LT882662	LT882666

*T* ex-type strain, *URM* URM Culture Collection (www.ufpe.br/micoteca), Brazil, *RB* personal working collection of Renan Barbosa, *CBS* culture collection of the Westerdijk Fungal Biodiversity Institute, The Netherlands

**Table 2:** Overview of isolated species from honey, bee pollen and nests

Species	Section	Isolate numbers	Honey	Pollen	Nests	Total
*Penicillium apimei* sp. nov.	*Gracilenta*	URM 7591 T= CBS 142502	1			1
*Penicillium brocae*	*Sclerotiora*	RB 001; RB 036; RB 035; RB 036; RB 046; RB 064; RB 075; RB 079; RB 082; RB 090; RB 093; RB 101; RB 116; RB 123; RB 124; RB 125; RB 181; RB 182; RB 184; RB 186; RB 193; RB 225	9		13	22
*Penicillium chermesinum*	*Charlesia*	RB 114			1	1
*Penicillium citreosulfuratum*	*Exilicaulis*	RB 094		1		1
*Penicillium citrinum*	*Citrina*	RB 006; RB 028; RB 032; RB 047; RB 068; RB 250; RB 086; RB 104; RB 109; RB 119; RB 134; RB 185; RB 187; RB 192; RB 198; RB 206	4	2	10	16
*Penicillium echinulonalgiovense* sp. nov.	*Lanata*-*Divaricata*	RB 201; RB 217; RB 218		1	2	3
*Penicillium fellutanum*	*Charlesia*	RB 112; RB 113			2	2
*Penicillium fernandesiae* sp. nov.	*Sclerotiora*	URM 7600 T = CBS 142500			1	1
*Penicillium mallochii*	*Sclerotiora*	RB 138; RB 151; RB 152			3	3
*Penicillium meliponae* sp. nov.	*Sclerotiora*	URM 7602 T = CBS 142495	1			1
*Penicillium mellis* sp. nov.	*Sclerotiora*	URM 7605 T= CBS 142499; URM 7611; RB 09; RB 69; RB 85; RB 110	2		4	6
*Penicillium paxilli*	*Citrina*	RB 127; RB 128			2	2
*Penicillium rubens*	*Chrysogena*	RB 014; RB 153; RB 161; RB 192 RB 210; RB 235			6	6
*Penicillium sanshaense*	*Sclerotiora*	URM 7617 T = CBS 142496		1		1
*Penicillium sclerotiorum*	*Sclerotiora*	RB 056; RB 121; RB 129; RB 237	1	1	2	4
*Penicillium shearii*	*Citrina*	RB 034; RB 073; RB 248			3	3
*Penicillium singorense*	*Lanata*-*Divaricata*	RB 202		1		1
*Penicillium* sp.	*Lanata*-*Divaricata*	URM 7610 = CBS 142497		1		1
*Penicillium steckii*	*Citrina*	RB 065; RB 088; RB 089; RB 137			4	4
*Penicillium sumatraense*	*Citrina*	RB 149			1	1
*Penicillium wotroi*	*Lanata*-*Divaricata*	RB 010; RB 158	1		1	2
*Talaromyces brasiliensis* sp. nov.	*Trachyspermi*	URM 7618 T = CBS 142493; URM 7619; URM 7620	1		2	3
*Talaromyces calidicanius*	*Talaromyces*	RB 183		1		1
*Talaromyces mycothecae* sp. nov.	*Talaromyces*	URM 7622 T = CBS 142494; URM 7623; RB 95; RB 171			4	4
*Talaromyces pigmentosus* sp. nov.	*Helici*	URM 7624 T= CBS 142805; URM 7625; RB 30; RB 96; RB 100		2	3	5
*Talaromyces scorteus*	*Islandici*	RB 072; RB 114; RB 148; RB 167	3		1	4
*Talaromyces wortmanii*	*Islandici*	RB 130			1	1
Total			23	11	66	100

*T* ex-type strain, *URM* URM Culture Collection (www.ufpe.br/micoteca), Brazil, *RB* personal working collection of Renan Barbosa, *CBS* culture collection of the Westerdijk Fungal Biodiversity Institute, The Netherlands

### Morphological analyses

For morphological analysis, the strains were three-point inoculated onto creatine agar (CREA), Czapek yeast extract agar (CYA), CYA supplemented with 5% NaCl (CYAS), dichloran 18% glycerol agar (DG18), malt extract agar (MEA, Oxoid), oatmeal agar (OA) and yeast extract sucrose agar (YES). All Petri dishes were incubated at 25 °C for 7 days and additional CYA and MEA plates were incubated at 15, 30 and 37 °C. Media preparation, inoculation and incubation were performed as described in Samson et al. (2010). Colony diameters were measured after 7 days of incubation and colony characteristics recorded (e.g. presence of soluble pigments, exudates, obverse and reverse colony colours, colour of mycelium). Microscopic observations of the asexual stage were made from colonies grown on MEA. The presence of a sexual stage was determined from cultures incubated on CYA, MEA and OA for at least 40 days at 25 °C. Lactic acid (60%) was used as mounting fluid and 96% ethanol was used to remove excess conidia. A Zeiss Stereo Discovery V20 dissecting microscope and a Zeiss AX10 Imager A2 light microscope, both equipped with Nikon DS-Ri2 cameras, were used to capture digital images using the software NIS-Elements D v4.50. The size, shape and pigmentation of microscopic features were recorded.

### DNA isolation, PCR and sequencing

Genomic DNA extractions were made from 7 days old colonies grown on MEA using the UltraClean Microbial DNA kit (MoBio Laboratories, Solana Beach, CA, USA). Polymerase chain reaction (PCR) amplification of the ITS barcode (ITS1, 5.8S rDNA and ITS2), *BenA*, *CaM* and *RPB2* gene regions were performed using methods described by Samson et al. (2010) and Houbraken et al. (2012). The PCR products were sequenced in both directions with the same primers using the BigDye^®^ Terminator v. 3.1 Cycle Sequencing Kit (Applied Biosystems Life Technologies, Carlsbad, CA, USA) and purified with Sephadex, according to the manufacturers’ recommendations. Contigs were assembled in the SeqMan (v.10.0.1; Madison, WI, USA) program using the forward and reverse sequence. Newly generated sequences were deposited in the NCBI nucleotide database (GenBank) and the European Nucleotide Archive (Table 1).

### Phylogenetic analysis

Sequence datasets were generated by combining the newly generated sequences with reference (preferably ex-type) sequences from NCBI (Visagie et al. 2014; Yilmaz et al. 2014; Taniwaki et al. 2015; Visagie et al. 2015; Chen et al. 2016; Laich and Andrade 2016; Luo et al. 2016; Romero et al. 2016; Rong et al. 2016; Visagie et al. 2016; Yilmaz et al. 2016; Guevara-Suarez et al. 2017; Wang et al. 2017a, b). The sequences were aligned using MAFFT v.7 (Katoh and Standley 2013) and manually optimized using MEGA v. 6.06 (Tamura et al. 2013). Individual alignments were concatenated by using Mesquite v. 3.04 (Maddison and Maddison 2016). The most suitable substitution model was determined using jModelTest v. 2.1.7 (Posada 2008). Phylogenetic trees were constructed using Maximum likelihood analyses (ML) using RAxML-HPC v. 8.2.8 (Stamatakis 2014) BlackBox with 1 000 rapid bootstrap inferences via the CIPRES science gateway (http://www.phylo.org/) (Miller et al 2010), while Bayesian inference (BI) analysis was performed in MrBayes 3.2.2 (Ronquist et al. 2012). In the Bayesian analyses, every 1 000 generations was sampled and the first 25% of the samples were discarded. Trees were visualized in FigTree v. 1.1.2 (Rambaut 2009) and edited in Adobe Illustrator v. 5.1. Bayesian inference (BI) posterior probabilities (pp) values and bootstrap (bs) values are labelled at the nodes. Values less than 0.95 pp and 70% bootstrap support are not shown. Branches with full support in Bayesian and RAxML analyses are thickened. Values below 0.95 pp and 70% are not shown and indicated with a hyphen. Aligned datasets and trees were uploaded to TreeBase (www.treebase.org) under submission number 21965.

### Extrolite analysis

Extrolites were extracted from the *Penicillium* strains after growing them on CYA, YES and MEA at 25 °C for 7 days. The *Talaromyces* strains were inoculated on CYA, YES, MEA and OA, and incubated at 25 °C for 14 days. Three agar plugs of each medium were extracted as previously described (Smedsgaard 1997; Houbraken et al. 2012). After extraction, the liquid was transferred to a clean screw-cap vial and evaporated to dryness. The dried extracts were re-dissolved in methanol by ultrasonication and filtered through a 0.45 µm filter. The extracts were analysed by ultra-high performance liquid chromatography with diode-array detection (UHPLC-DAD) (Houbraken et al. 2012). The detected eluted compounds were identified by comparison of the retention time, retention index and the UV spectrum measured at 200–600 nm against UV spectra from made from standards and data from literature (Nielsen et al. 2011; Klitgaard et al. 2014).

## Results

### Isolation and identification

During this study on the fungal diversity of substrates related to stingless bees, isolates belonging to various genera [e.g. *Aspergillus*, *Fusarium*, *Monascus* (Barbosa et al. 2017), *Penicillium*, *Talaromyces*] were isolated. This study focusses on the identification of the detected *Penicillium* and *Talaromyces* diversity. The number of *Talaromyces* species (and isolates) detected during this study is low compared to *Penicillium*. Eighty-two *Penicillium* and 18 *Talaromyces* isolates were obtained during the survey on fungi present in honey, bee pollen and inside the nests of *Melipona scutellaris* bees. Phenotypic characters, combined with ITS and partial *BenA* sequences were used to identify isolates. In total, 21 *Penicillium* and six *Talaromyces* species were present among the investigated isolates. Among those, five *Penicillium* and three *Talaromyces* species displayed unique characters deviating from known species. Seven of those eight species are described here as new (see Taxonomy section), and one isolate (RB115), belonging to section *Lanata*-*Divaricata*, will be described elsewhere. Three new *Penicillium* species belong to section *Sclerotiora* and one to section *Gracilenta*; the three new *Talaromyces* are classified in sections *Helici, Talaromyces* and *Trachyspermi*. An overview of the species isolated during this study is given in Table 2. The highest *Penicillium* and *Talaromyces* occurrence frequency was observed in the samples collected from the inside of nests (66%). The majority of the isolated *Penicillium* species belonged to sections *Sclerotiora* (46%) and *Citrina* (30%). *Penicillium brocae* was most frequently isolated (22%), followed by *Penicillium citrinum* (16%), *Penicillium rubens* (6%) and *Penicillium mellis* sp. nov. (6%).

### Phylogeny

The phylogenetic relationship of the new *Penicillium* and *Talaromyces* species with accepted species was determined by analysis of single and concatenated sequence datasets of three or four loci (ITS, *BenA*, *CaM* and/or *RPB2*). An overview of the length of each dataset and the most optimal substitution model is given Table 3. The multigene phylograms are show in the manuscript and the single gene trees in Supplementary data.

**Table 3:** Sequence data sets and models used in the phylogenetic analyses

Section	ITS (bp)	Substitution model	*BenA* (bp)	Substitution model	*CaM* (bp)	Substitution model	*RPB2* (bp)	Substitution model
*Penicillium* sect. *Gracilenta*	493	TrN+G	444	GTR+G	570	K80+G	895	TrN+G
*Penicillium* sect. *Lanata*-*Divaricata*	500	GTR+G	443	GTR+G	499	GTR+G	755	GTR+G
*Penicillium* sect. *Sclerotiora*	536	GTR+G	406	GTR+G	456	TrN+G	n/a	n/a
*Talaromyces* sect. *Helici*	464	HKY+G	432	HKY+G	564	TrN+G	852	TrN+G
*Talaromyces* sect. *Talaromyces*	459	TrN+G	397	HKY+G	515	TrN+G	706	HKY+G
*Talaromyces* sect. *Trachyspermi*	472	GTR+G	394	TrN+G	515	K80+G	517	GTR+G

*n/a* not available

#### *Penicillium* section *Gracilenta*

**Fig. 1:**
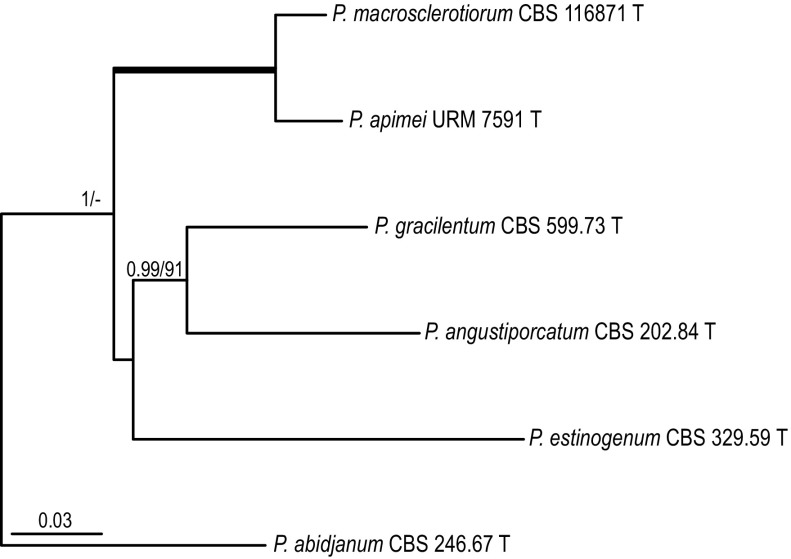
Phylogeny based on the combined ITS, *BenA*, *CaM* and *RPB2* data set for species classified in *Penicillium* section *Gracilenta. Penicillium abidjanum* CBS 246.67 was chosen as outgroup

Section *Gracilenta* contains four species, *P. angustiporcatum*, *P. estinogenum*, *P. gracilentum* and *P. macrosclerotiorum*. *Penicillium apimei* sp. nov. is in all phylogenies, with high statistical support (> 0.95 pp, > 70% bs), related to *P. macrosclerotiorum* (Fig. 1). ITS, *BenA* and *CaM* sequences can distinguish all species in this section.

#### *Penicillium* section *Lanata*-*Divaricata*

**Fig. 2:**
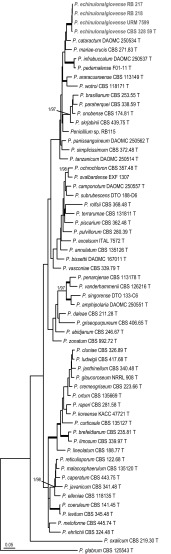
Phylogeny based on the combined ITS, *BenA* and *CaM* data set for species classified in *Penicillium* section *Lanata*-*Divaricata*. *Penicillium glabrum * CBS 125543 was chosen as outgroup

Isolates URM 7599, RB 217 and RB 218 cluster together in all phylograms, and always close to *P. echinulonalgiovense* CBS 328.59. The *BenA* phylogeny shows that these three isolates and *P. echinulonalgiovense* CBS 328.59 are related with full support to *P. cataractum* DAOMC 250534. The *CaM*, ITS, *RPB2* and combined phylogenies could not resolve the phylogenetic relationship of these isolates (Fig. 2, Suppl. Figures 2, 3).

#### *Penicillium* section *Sclerotiora*

**Fig. 3:**
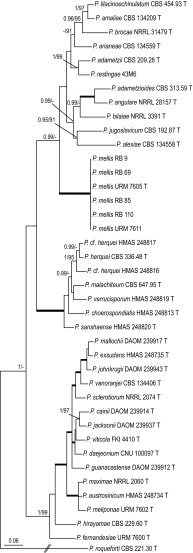
Phylogeny based on the combined ITS, *BenA*, and *CaM* data set for species classified in *Penicillium* section *Sclerotiora*. *Penicillium glabrum* CBS 125543 was chosen as outgroup

Isolate URM 7602^T^ (*Penicillium meliponae* sp. nov.) resides in a well-supported clade with *P. maximae* NRRL 2060^T^ and *P. austrosinicum* HMAS 248734^T^ (ITS: 0.99 pp, 99% bs; *BenA*: 1.00 pp, 100% bs; *CaM*: 1.00 pp, 93% bs). *Penicillium fernandesiae* sp. nov. (URM 7600^T^) clusters with *P. hirayamae* CBS 229.60^T^ in our ITS (< 0.95 pp, 91% bs) phylogram. Analysis of the *BenA*, ITS and combined dataset shows that this species belongs to a large clade containing e.g. *P. sclerotiorum*, *P. maximae* and *P. hirayamae*, the so-called *P. sclerotiorum*-clade. Isolates URM 7605^T^, URM 7611, RB 9, RB 69, RB 85 and RB 110 resolved in all analyses in a single, distinct, well-supported branch and are described here as *P. mellis* sp. nov. Analysis of *BenA* and ITS sequences could not resolve the phylogenetic position of *P. mellis* sp. nov. in section *Sclerotiora*. This species has, in the *CaM* phylogram, a basal position to a clade containing e.g. *P. bilaiae*, *P. brocae* and *P. adametzioides*. *Penicillium mellis* sp. nov. takes a basal position to *P. bilaiae* and related species in the phylogeny based on a combined dataset of ITS, *BenA* and *CaM* sequences (Fig. 3). This relationship is supported with a high posterior probability value (0.99), but a low bootstrap percentage (< 70%). A limited number of *RPB2* sequences are available for section *Sclerotiora* and therefore no phylogenetic analysis was performed for this locus.

#### *Talaromyces* section *Helici*

**Fig. 4:**
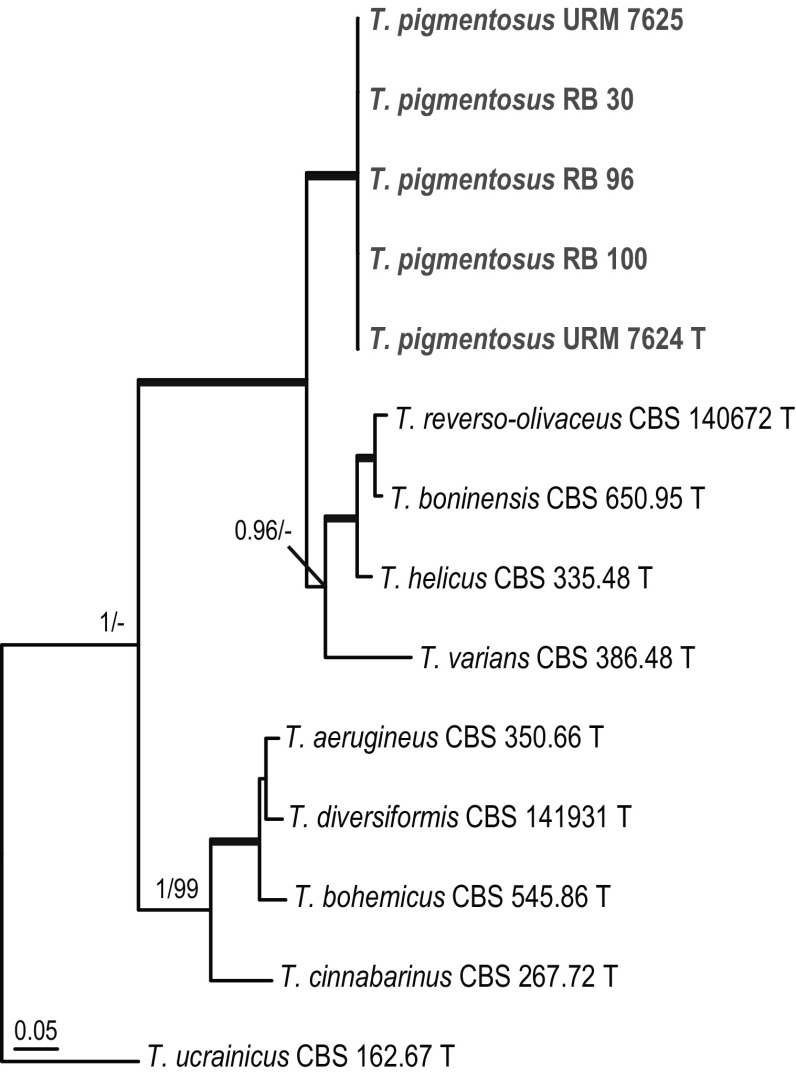
Phylogeny based on the combined ITS, *BenA*, *CaM* and *RPB2* data set for species classified in *Talaromyces* section *Helici. Talaromyces ucrainicus* CBS 162.67 was chosen as outgroup

Nine species are currently accepted in section *Helici*. The combined phylogenetic analysis (Fig. 4) revealed the presence of two well supported clades. One clade contained the species *T. reverso*-*olivaceus*, *T. helicus*, *T. georgiensis, T. boninensis* and *T. varians* (clade 1) and the other *T. aerugineus*, *T. diversiformis*, *T. bohemicus* and *T. cinnabarinus* (clade 2). Five strains isolated during this study clustered together in all (single gene) phylogenies and are here described as a new species named *Talaromyces pigmentosus*. *Talaromyces pigmentosus* sp. nov. clusters in clade 1 with *T. reverso*-*olivaceus*, *T. helicus*, *T. boninensis* and *T. varians*. The combined analysis showed, with high statistical support, that the *T. pigmentosus* sp. nov. isolates have a basal position to these clade 1 members (Fig. 4).

#### *Talaromyces* section *Talaromyces*

**Fig. 5:**
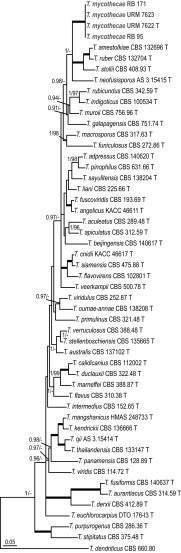
Phylogeny based on the combined ITS, *BenA*, *CaM* and *RPB2* data set for species classified in *Talaromyces* section *Talaromyces*. *Talaromyces dendriticus* CBS 660.80 was chosen as outgroup

The phylogenetic relationship of *T. mycothecae* sp. nov. is difficult to determine based on the single gene phylogenies (Suppl. Figures 7, 8). In the *BenA* analysis, the species is close to *T. neofusisporus, T. amestolkiae*, *T. ruber*, *T. stollii* (0.99 pp, < 70% bs) and the species is in the *CaM* and *RPB2* phylogenies close to *T. ruber*, *T. amestolkiae* and *T. stolii*, though with poor or no support (*CaM* < 0.95 pp, < 70% bs; *RPB2* 1.00 pp, < 70% bs). The phylogenetic relationship based on the BI analysis of the combined dataset indicated a relationship with *T. neofusisporus, T. amestolkiae*, *T. ruber* and *T. stollii* (1.00 pp); however, no statistical support in the ML analysis was found (< 70% bs) (Fig. 5).

#### *Talaromyces* section *Trachyspermi*

**Fig. 6:**
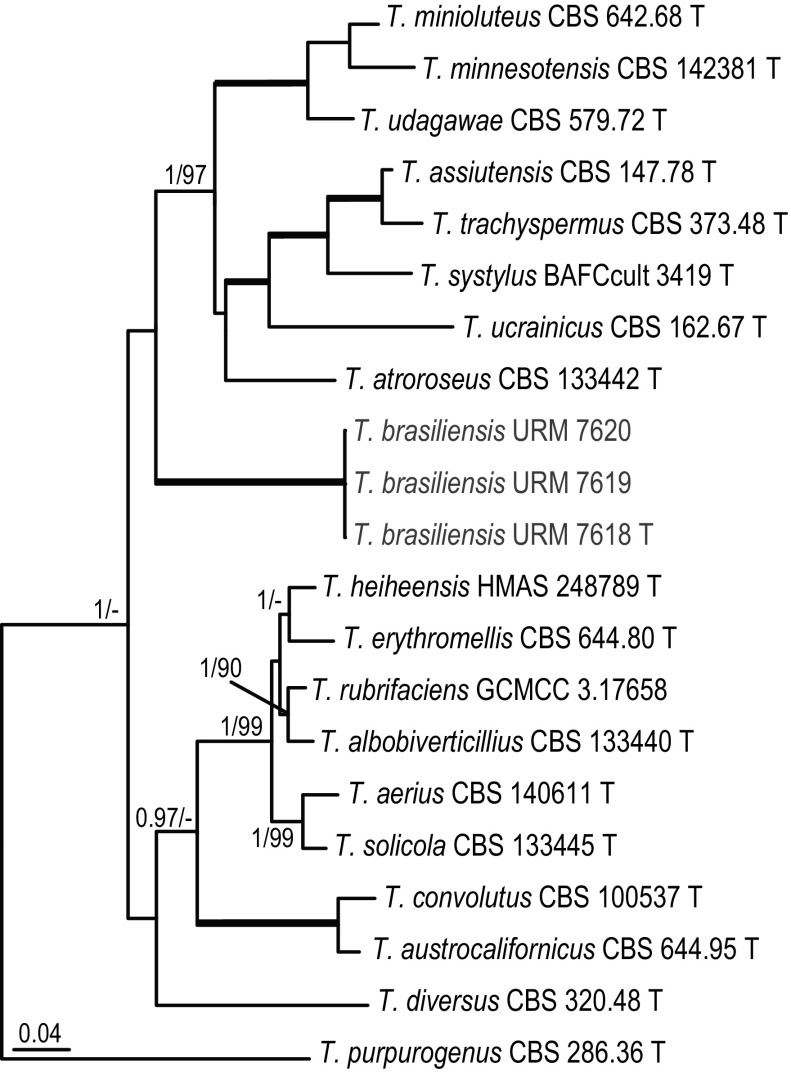
Phylogeny based on the combined ITS, *BenA*, *CaM* and *RPB2* data set for species classified in *Talaromyces* section *Trachyspermi. Talaromyces purpurogenus* CBS 286.36 was chosen as outgroup

Isolates URM 7618^T^, URM 7619 and URM 7620 formed a clade together in all analyses. This set of isolates is described here as a new species named *T*. *brasiliensis*. The phylogenetic relationship of this species with other members of this section is unknown. The analysis of the combined dataset indicates that this species is basal to *T. assistuensis*, *T. atroroseus*, *T. minioluteus, T. systylus*, *T. trachyspermus*, *T. ucrainicus* and *T. udagawae*, but statistical support is lacking (< 0.95 pp, < 70% bs) (Fig. 6).

### Extrolites

The majority of investigated *Penicillium* and *Talaromyces* species were producers of different kinds of extrolites. An overview of results is given in Table 4. *Penicillium apimei* sp. nov. produced spinulosin, four members of the geodin biosynthetic family (asterric acid, erdin, geodin, sulochrins) and an uncharacterized compound belonging to “biosynthetic family G”. The new species in section *Sclerotiora* produced sclerotiorins, patulodin (or similar) and kojic acid. Our fresh isolate of *P. echinulonalgiovense* (sect. *Lanata*-*Divaricata*) produced xanthoepocin and andrastin A. *Talaromyces mycothecae* produced duclauxin, a compound with a rubropunctatin chromophore and various extrolites also produced by other members of section *Talaromyces*. The new species *T. pigmentosus* (sect. *Helici*) and *T. brasiliensis* (sect. *Trachyspermi*) produced several uncharacterized extrolites that appear to be unique for the species.

**Table 4:** Extrolites detected in the investigated *Penicillium* and *Talaromyces* species

Species	Strain examined	Extrolites
*Penicillium apimei*	URM 7591 T = CBS 142502	Asterric acid, (−)-bisdechlorogeodin, erdin, geodin, spinulosin X, sulochrin
*Penicillium brocae*	RB 075; RB 125	Brocaenol, pyranonigrin F, spinulosin X
*Penicillium chermesinum*	RB 144	Extrolites with end-absorbtion
*Penicillium citreosulfuratum*	RB 094	Citroviridin; pyrenocins
*Penicillium citrinum*	RB 028; RB 059	Citrinin; quinolactacin; citrinadin
*Penicillium echinulonalgiovense*	RB 201	Andrastin A, xanthoepocin
*Penicillium fernandesiae*	URM 7600 T = CBS 142500	Rotiorin, sclerotiorin and other members of the sclerotiorin biosynthetic family
*Penicillium mallochii*	RB 151; RB 152	Atlantinone A
*Penicillium meliponae*	URM 7602 T = CBS 142495	Rotiorin, sclerotiorin and other members of the sclerotiorin biosynthetic family
*Penicillium mellis*	URM 7605 T = CBS 142499; URM 7611	Kojic acid; Kojic acid and sclerotiorin
*Penicillium fellutanum*	RB 112; RB 113	Many extrolites with end absorbtion
*Penicillium paxilli*	RB 127; RB 128	Pyrenocine; paxillin; paspaline; paspalinine
*Penicillium* sp.	RB 115	Atlantinone A, fumitremorgin A, B & C, verruculogen
*Penicillium rubens*	RB 014; RB 153	Andrastin A; glandicolins; roquefortine C; meleagrin; chrysogine, meleagrin, roquefortine C, sorbicillins
*Penicillium sanshaense*	URM 7617 T = CBS 142496	Atrovenetin, emodin, an emodin bisanthron, naphthalic anhydride, members of the herqueinone biosynthetic family
*Penicillium sclerotiorum*	RB 056; RB 237	Extrolite with orthosporin chromophore, rotiorin, sclerotiorin and other related extrolites
*Penicillium shearii*	RB 034; RB 073	Indole alkaloids; paspaline; paxillin; shearinins with an extended chromophore
*Penicillium singorense*	RB 202	Special shearinins, paspaline or paspaline-like
*Penicillium steckii*	RB 065; RB 088	Isochromantoxin; quinolactacin
*Penicillium sumatrense*	RB 149	Curvularin; daldinins
*Penicillium wotroi*	RB 010	Xanthoepocin
*Talaromyces brasiliensis*	URM 7618 T = CBS 142493; URM 7619	Many extrolites detected, none of them could be identified, and none of them have been observed in other *Talaromyces* or *Penicillium* species before.
*Talaromyces calidicanius*	RB 183	duclauxin and other members of the duclauxin biosynthetic family
*Talaromyces mycothecae*	URM 7622 T = CBS 142494; URM 7623	Duclauxin and other duclauxins, compound with a rubropunctatin chromophore, many further extrolites detected, none of them could be identified, and none of them have observed in other *Talaromyces* or *Penicillium* species before.
*Talaromyces pigmentosus*	URM 7624 T= CBS 142805; URM 7625	Many extrolites detected, none of them could be identified, and none of them have been observed in other *Talaromyces* or *Penicillium* species before.
*Talaromyces scorteus*	RB 072; RB 114	Rugulosin and skyrin detected in, several unknown extrolites
*Talaromyces wortmannii*	RB 130	Rugulovasine A; rugulosin; skyrin; ukulactones

*T* ex-type strain, *URM* URM Culture Collection (www.ufpe.br/micoteca), Brazil, *RB* personal working collection of Renan Barbosa, *CBS* culture collection of the Westerdijk Fungal Biodiversity Institute, The Netherlands

## Taxonomy

**Fig. 7:**
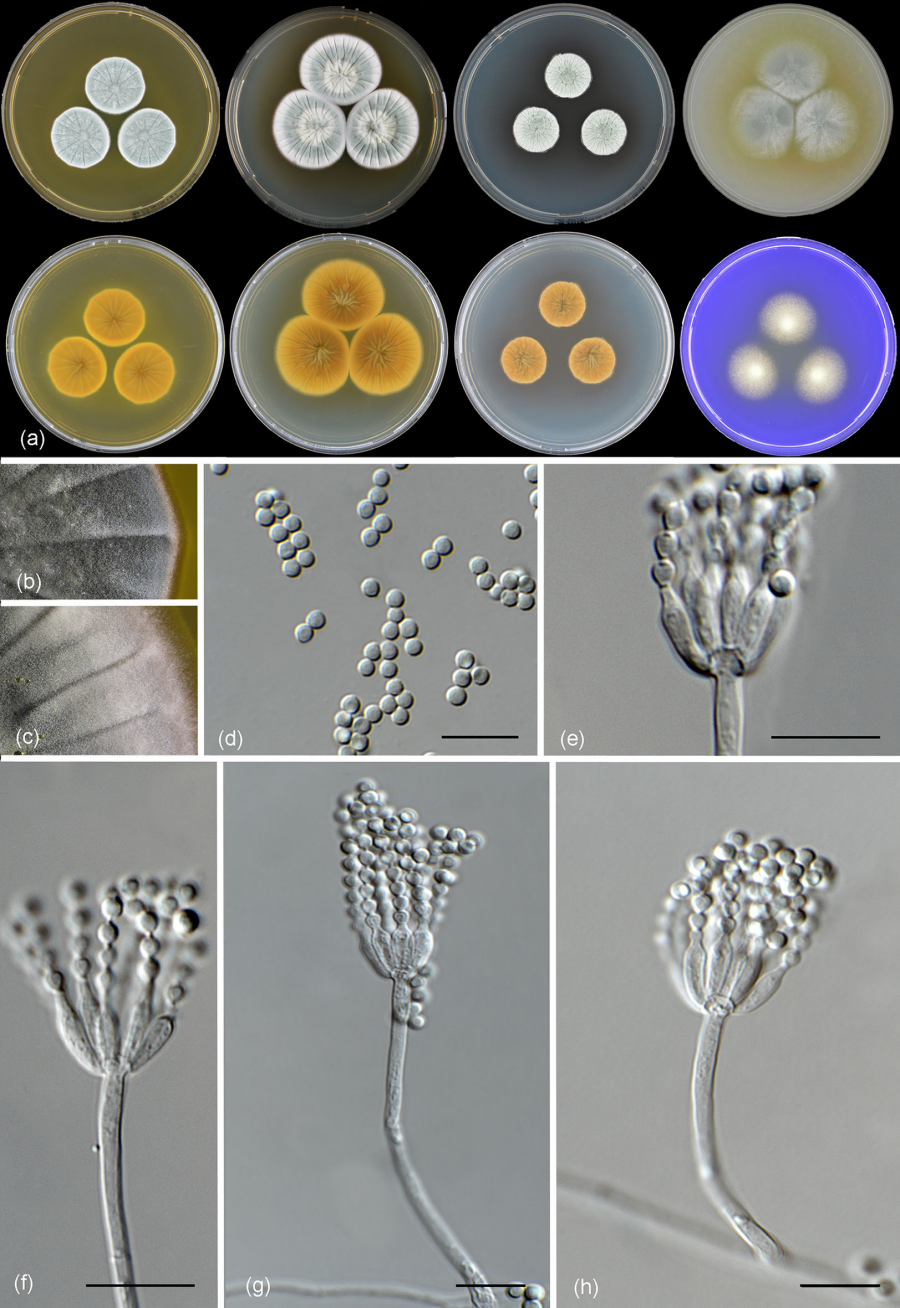
Morphological characters of *Penicillium apimei* CBS 142502. **a** Colonies from left to right (top row) MEA, CYA, YES and OA; (bottom row) CYA reverse, MEA reverse, YES reverse and CREA. **b** Texture on CYA. **c** Texture on MEA. **d** Conidia. **e–h** Conidiophores. Scale bars 10 μm

*Penicillium apimei* R.N. Barbosa, Souza-Motta, N.T. Oliveira & Houbraken sp. nov. (Figure 7)

MycoBank: MB 822208

*Etymology*: *apimei* refers to APIME, the stingless beekeeping association in Pernambuco, Brazil, which gave support for collecting samples used for this study.

*Diagnosis*: *Penicillium apimei* sp. nov. belongs to section *Gracilenta* and is phylogenetically unique. The species is strictly monoverticillate, grows well on MEA and CYA at 25 °C and is able to grow 37 °C.

*Type*: Brazil: *Pernambuco*: Recife, from honey of *Melipona scutellaris*, April 2014, *R.N. Barbosa*. Holotype (slide preparation) is deposited in the URM Mycology Herbarium (Recife, Brazil): URM 90489; ex-type strains URM 7591 = CBS 142502.

*ITS barcode*: MF278310. Alternative markers: *BenA* = LT854641; *CaM* = LT882717; *RPB2* = LT854650.

*Colony diam, 7* *days* (*in mm*): CYA 29–31; CYA 15 °C 10–12; CYA 30 °C 40–41; CYA 37 °C 7–9; MEA 25–27; MEA 15 °C 14–15; MEA 30 °C 39–40; MEA 37 °C 6–8; DG18 22–23; CYAS 20–23; OA 29–30; YES 38–40; CREA 22–23.

*Colony characters*: CYA, 25 °C, 7 days: Colonies moderately deep, radially sulcate; margins entire, low, narrow; mycelium white; colony texture velvety; sporulation moderate; conidial colour *en masse* greyish green; exudate clear to yellowish; soluble pigment yellow amber to brownish; reverse brown. MEA, 25 °C, 7 days: Colonies plane, slightly raised at centre, radially sulcate; margins entire, low, narrow; mycelium white sometimes inconspicuously grey; colony texture velvety to floccose; sporulation moderate to strong; conidial colour *en masse* greyish green; exudate present as small clear droplets; soluble pigment absent, reverse brownish. YES, 25 °C, 7 days: Colonies moderately deep, radially and concentrically sulcate; margins low, narrow, entire; mycelium white to grey; colony texture velvety; sporulation moderate to strong, conidia *en masse* greyish green; exudate absent; soluble pigment yellow; reverse yellow to brownish elsewhere. DG18, 25 °C, 7 days: Colonies plane, raised at centre; margins low, entire; mycelium white; colony texture velvety; sporulation moderate; conidial colour *en masse* greyish green; exudate absent; soluble pigment absent; reverse yellow, sometimes inconspicuously greenish. OA, 25 °C, 7 days: Colonies flat, entire; margins regular; mycelium white to inconspicuously yellow; colony texture velvety; sporulation sparse; conidial colour *en masse* greyish green; exudate absent; soluble pigment yellow; reverse yellowish to cream. CYAS 25 °C, 7 days: Colonies plane, raised at centre, radially and concentrically sulcate; margins low, narrow, entire; mycelium white; colony texture velvety; sporulation sparse, conidial colour *en masse* greyish; exudate absent; soluble pigment brownish; reverse brown. CREA, 25 °C, 7 days: good growth, acid production absent.

*Micromorphology*: Conidiophores strictly monoverticillate. Stipes smooth walled, 25–90 × 1–4.5 μm, vesiculate, up to 4 µm in diam. Phialides 4–10 per stipe, ampulliform, 6.5–9.5 × 2.0–3.0 μm. Conidia smooth walled, globose, 2.0–3.0 × 2.0–3.0 μm. Sclerotia or ascomata not observed.

*Notes*: Houbraken and Samson (2011) did not report any significant similarities shared between species belonging to section *Gracilenta*, except that all species weren’t able to grow at 37 °C and had brown reverses on Czapek agar or CYA. The reverse colony colour of *P. apimei* on CYA and YES is also in shades of brown, but the species is unique in for its ability to grow at 37 °C. This species is phylogenetically most closely related to *P. macrosclerotiorum*. Besides its ability to grow at 37 °C, it can further be differentiated from this species by the absence of sclerotia and slower growth on YES (38–40 vs 54–56 mm).

*Penicillium echinulonalgiovense* S. Abe ex Houbraken & R.N. Barbosa sp. nov.

MycoBank: MB822213

= *Penicillium echinulonalgiovense* S. Abe, Journal of General and Applied Microbiology 2: 80. 1956. [MB536546]. (nom. inval., Art. 39.1.).

*Diagnosis*: *Penicillium echinulonalgiovense* sp. nov. is phylogenetically unique. Colonies on CYA incubated at 25 °C for 7 days attain a diameter of 33–37 mm and on CYA 37 °C 8–12 mm. The growth on CREA is weak, the colony diameter 23–27 mm, and no acid compounds are produced. The conidiophore stipes are rough walled, and conidia are globose to subglobose and echinulate.

*Type*: Japan: unrecorded source, *S. Abe*. Holotype: CBS H–23172; ex-type strains CBS 328.59 = ATCC 18314 = FAT 907 = FRR 638 = IFO 6229 = IMI 068213 = QM 7301.

*ITS barcode*: GU981587. Alternative markers: *BenA* = GU981631; *CaM* = KX961269; *RPB2* = KX961301.

*Additional material examined*. Australia, Atherton Tableland, Queensland, soil, *R. van Leeuwen & J. Houbraken*, DTO 030-D8; China, Hong Kong, soil, isol. by *W. Gams & A. Aptroot*, CBS 102417; Indonesia, Yogyakarta, storage room, DTO 232-C6; Netherlands, industrial installation, *J. Houbraken*, CBS 115322; Madagascar, Ifaty, forest soil, coll. *F. Hagen,* isol. *J. Houbraken*, DTO 088-A2; Malaysia, Langkawi, soil of rainforest, coll. *R.A. Samson,* isol. *J. Houbraken*, DTO 054-A1; USA, Florida, soil from citrus grove, *R.A. Samson*, DTO 010-A5. Brazil, Bee pollen of *Melipona scutellaris* URM 7599; inside of nests of *Melipona scutellaris* RB 217; RB 218 coll. R.N.Barbosa.

*Notes*: *Penicillium echinulonalgiovense* was described without a Latin diagnosis. To validate the species, an English diagnosis is given above, with the name of the original author maintained. The ITS and partial *BenA* and *CaM* sequence data had sufficient discriminatory power to differentiate *P. echinulonalgiovense* (CBS 328.59^T^) from *P. simplicissimum* and other described species in section *Lanata*-*Divaricata*. In the *BenA* analysis (Suppl. Figure 2), *P. echinulonalgiovense* is related to *P. cataractum* DAOMC 250534^T^ and *P. mariae*-*crucis* (CBS 271.83^T^). *Penicillium echinulonalgiovense* can be differentiated from those species by its ability to grow on CYA incubated at 37 °C (8–12 mm). Furthermore, *P. cataractum* grows moderately well on CREA and produces high levels of acid compounds on this medium. Both *P. echinulonalgiovense* and *P. mariae*-*crucis* grow poorly on CREA and do not produce acid compounds. In addition, the reverse colours on CYA differ. The reverse colour of *P. echinulonalgiovense* on CYA is dark brown in the centre and beige towards the margins, the reverse colour of *P. mariae*-*crucis* is blackish brown and those of *P. cataractum* greyish yellow to greyish orange (Visagie et al. 2016).

**Fig. 8:**
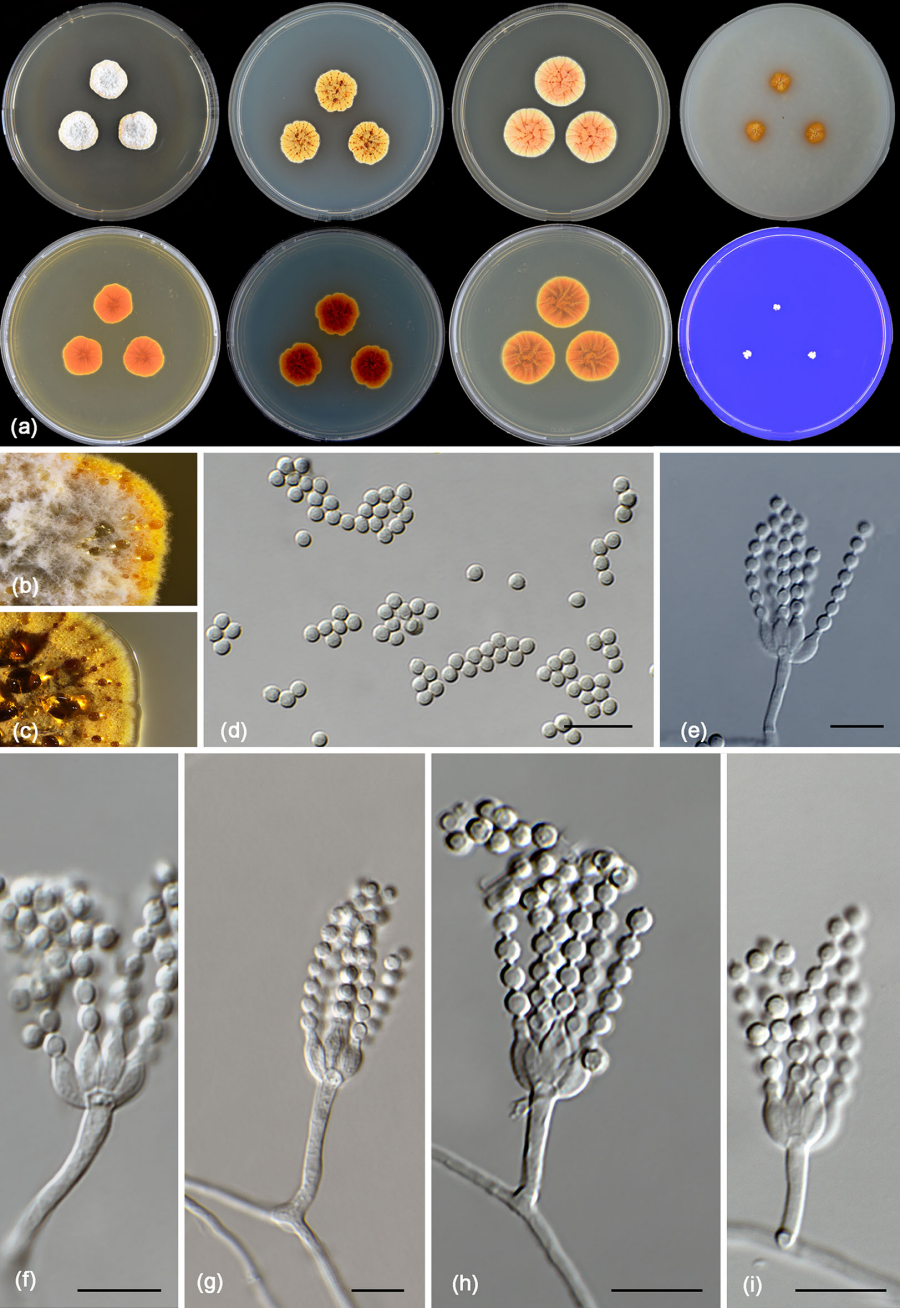
Morphological characters of *Penicillium fernandesiae* CBS 142500. **a** Colonies from left to right (top row) MEA, CYA, YES and OA; (bottom row) CYA reverse, MEA reverse, YES reverse and CREA. **b** Texture on CYA. **c** Texture on MEA. **d** Conidia **e–i** Conidiophores. Scale bars 10 μm

*Penicillium fernandesiae* R.N. Barbosa, Souza-Motta, N.T. Oliveira & Houbraken sp. nov. (Figure 8)

MycoBank: MB822209

*Etymology*: Named in honour of prof. Maria José Fernandes, mycologist working with *Aspergillus* and *Penicillium* in the former Institute of Mycology of the University of Recife (IMUR), Pernambuco, Brazil.

*Diagnosis*: Red soluble pigments produced on CYA, no growth on MEA and CYA at 37 °C, restricted growth on CYA, MEA, YES, CYAS and no acid production on CREA.

*Type*: Brazil: *Pernambuco*: Recife, inside nests of *Melipona scutellaris*, May 2014, *R.N. Barbosa*. Holotype (slide preparation) is deposited in the URM Mycology Herbarium (Recife, Brazil): URM 90490; ex-type strains URM 7600 = CBS 142500.

*ITS barcode*: MF278314. Alternative markers: *BenA* = LT854645; *CaM* = LT854649; *RPB2* = LT854654.

*Colony diam, 7* *days* (*in mm*): CYA 15–18; CYA15 °C 5–6; CYA30 °C 20–22; CYA37 °C no growth; MEA 15–17; MEA 15 °C 4–5; MEA 30 °C 20–22; MEA 37 °C no growth; DG18 17–18; CYAS 15–17; OA 6–8; YES 21–22; CREA 3–4.

*Colony characters*: CYA, 25 °C, 7 days: Colonies moderately deep, gently radially sulcate; margins low, undulate, entire; mycelium yellow; colony texture velvety to floccose; sporulation absent; conidial colour *en masse* indeterminable; exudate orange; soluble pigment in shades of red; reverse orange to brownish at centre. MEA, 25 °C, 7 days: Colonies convex; margins low, narrow, entire; mycelium white, sometimes inconspicuously yellow; colony texture floccose; sporulation absent; conidial colour *en masse* indeterminable; exudate clear at centre and sometimes orange close the margins; soluble pigment absent; reverse orange. YES, 25 °C, 7 days: Colonies moderately deep, radially and concentrically sulcate; margins low, narrow, entire; mycelium white to slightly inconspicuously yellow; colony texture floccose; sporulation absent; conidial colour *en masse* indeterminable; exudate orange; soluble pigment absent; reverse brownish to orange. DG18, 25 °C, 7 days: Colonies moderately deep, gently radially sulcate; margins low, narrow, entire; mycelium yellow, texture velvety to floccose; sporulation absent; conidial colour *en masse* indeterminable; exudate orange; soluble pigment absent; reverse orange. OA, 25 °C, 7 days: Colonies flat, margins irregular; mycelium yellow; sporulation absent, conidial colour *en masse* indeterminable; exudate clear orange; soluble pigment absent; reverse orange. CYAS 25 °C, 7 days: Colonies moderately deep, radially and concentrically sulcate; margins low, narrow, entire; mycelium white; colony texture floccose; sporulation absent; conidial colour *en masse* indeterminable; exudate orange; soluble pigment absent; reverse brownish orange. CREA, 25 °C, 7 days: Very weak growth, acid production absent.

*Micromorphology*: Conidiophores strictly monoverticillate. Stipes smooth walled, 7.5–20 × 1.5–2.0 μm, non-vesiculate. Phialides 4–7 per stipe, ampulliform, 6–11 × 2.0–3.0 μm. Conidia smooth walled, globose, 2–3 μm. Sclerotia or ascomata not observed.

*Notes*: *Penicillium fernandesiae* sp. nov. belongs to the *P. sclerotiorum*-clade. The species produces sclerotiorins and these compounds are shared with *P. hirayamae*, *P. meliponae* and *P. sclerotiorum* and other species in the *P. sclerotiorum*-clade, which is in line with its phylogenetic placement. *Penicillium fernandesiae* produces red soluble pigments on CYA and these are not produced by the closely related species *P. hirayamae*. Red soluble pigment production is shared with *P. adametzioides*, a phylogenetically distant species (Visagie et al. 2013).

**Fig. 9:**
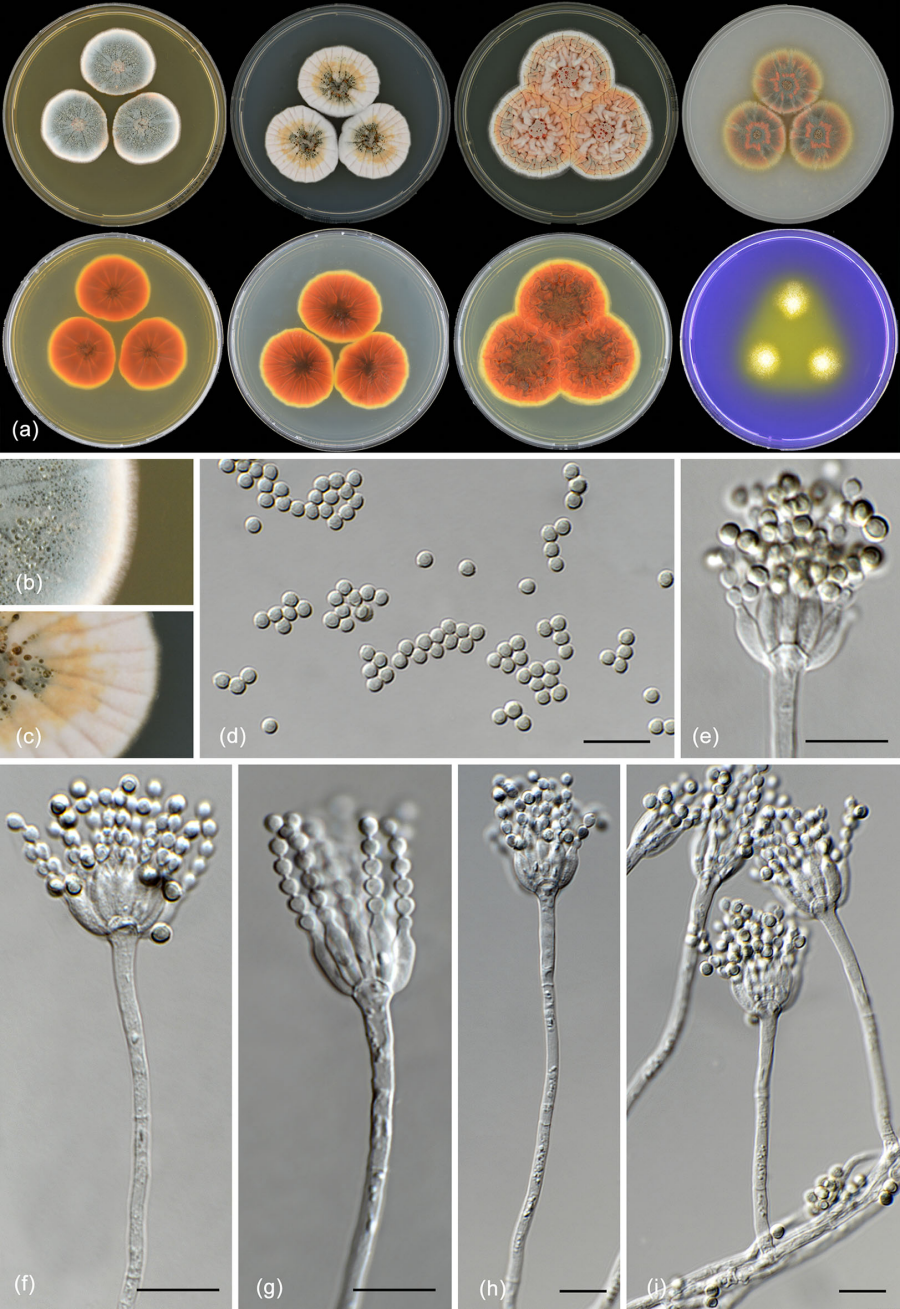
Morphological characters of *Penicillium meliponae* CBS 142495. **a** Colonies from left to right (top row) MEA, CYA, YES and OA; (bottom row) CYA reverse, MEA reverse, YES reverse and CREA. **b** Texture on CYA. **c** Texture on MEA. **d** Conidia. **e–i** Conidiophores. Scale bars 10 μm

*Penicillium meliponae* R.N. Barbosa, Souza-Motta, N.T. Oliveira & Houbraken sp. nov. (Figure 9)

MycoBank: MB822210

*Etymology*: *meliponae*, refers to *Melipona scutellaris*, the stingless bee species investigated in this study.

*Diagnosis*: *Penicillium meliponae* sp. nov. have colony diameter on CYA, MEA, DG18, CYAS and CREA generally below to 32 mm. The species grows moderately well on CREA and has a strong acid production.

*Type*: Brazil: *Pernambuco*: Recife, honey of *Melipona scutellaris*, June 2014, *R.N. Barbosa*. Holotype (slide preparation) is deposited in the URM Mycology Herbarium (Recife, Brazil): URM 90491; ex-type strains URM 7602 = CBS 142495.

*ITS barcode*: MF278315. Alternative markers: *BenA* = LT854644; *CaM* = LT854648; *RPB2* = LT854653.

*Colony diam, 7* *days* (*in mm*): CYA 30–32; CYA15 °C 15–16; CYA30 °C 25–28; CYA37 °C no growth; MEA 30–31; MEA 15 °C 9–10; MEA 30 °C 24–25; MEA 37 °C no growth; DG18 25–26; CYAS 23–25; OA 26–28; YES 40–43; CREA 17–18.

*Colony characters*: CYA, 25 °C, 7 days: Colonies radially sulcate, slightly raised at centre; margins low, narrow, entire; mycelium white; colony texture floccose; sporulation absent at margin, strong in centre; conidial colour *en masse* greyish green; exudate orange; soluble pigment orange; reverse brown at centre, orange at the margins and yellow at the borders. MEA, 25 °C, 7 days: Colonies plane, moderately deep, lightly radially sulcate; margins entire, low, narrow, entire; mycelium white and slightly orange; colony texture floccose at centre somewhat velvety in some areas close the margins; sporulation strong in centre, weak at margins; conidial colour *en masse* greyish; exudate hyaline to pale orange; soluble pigment absent; reverse dull orange. YES, 25 °C, 7 days: Colonies moderately deep, raised at centre, randomly sulcate; margins low, narrow, entire; mycelium white, sometimes inconspicuously orange; colony texture floccose; sporulation sparse; conidial colour *en masse* greyish in some areas; exudate orange; soluble pigment absent; reverse reddish brown at centre fading to orange close to margin and yellow in the borders. DG18, 25 °C, 7 days: Colonies moderately deep, lightly sulcate; margins entire; mycelium white; colony texture floccose to velvety; sporulation sparse; conidial colour *en masse* indeterminable; exudate orange; soluble pigment absent; reverse orange at centre to yellow in the margins. OA, 25 °C, 7 days: Colonies plane, not sulcate; margins entire; mycelium yellow, sometimes white; colony texture velvety, sporulation sparse, conidial colour somewhat greyish, exudate orange, in small droplets; soluble pigment absent; reverse orange at centre to yellow at the margins. CYAS, 25 °C, 7 days: Colonies slightly raised, radially and concentrically sulcate; margins low, narrow, entire; mycelium white; colony texture floccose; sporulation absent to moderate at centre; conidial colour *en masse* greyish; exudate orange, soluble pigment absent; reverse reddish brown at centre fading to orange close to margin and yellow at the borders. CREA, 25 °C, 7 days: Moderate growth; acid produced.

*Micromorphology*: Conidiophores strictly monoverticillate. Stipes smooth walled 22.0–45 × 2.5–3.5 μm, vesiculate 4.0–6.5 μm. Phialides 4–12 per stipe, ampulliform, 6.0–9.0 × 2.5–4.0 μm. Conidia smooth walled, subglobose, 2.0–3.0 μm. Sclerotia not observed produced.

*Notes*: *Penicillium meliponae* sp. nov. is phylogenetically most closely related to *P. maximae* and *P. austrosinicum*. *Penicillium meliponae* sp. nov. produces smaller colonies on CYA, MEA, CYAS and CREA after 7 days incubation at 25 °C than *P. austrosinicum* and *P. maximae*. Furthermore, *P. meliponae* sp. nov. has a strong acid production on CREA, while *P. maximae* lacks acid production on CREA (Visagie et al. 2013). *Penicillium austrosinicum* produces subglobose, rough walled conidia, the conidia of *P. meliponae* sp. nov. are subglobose and smooth and those of *P. maximae* are ellipsoidal and smooth. Additionally, *P. meliponae* sp. nov. and *P. maximae* do not produce sclerotia, while *P. austrosinicum* does (Wang et al. 2017a).

**Fig. 10:**
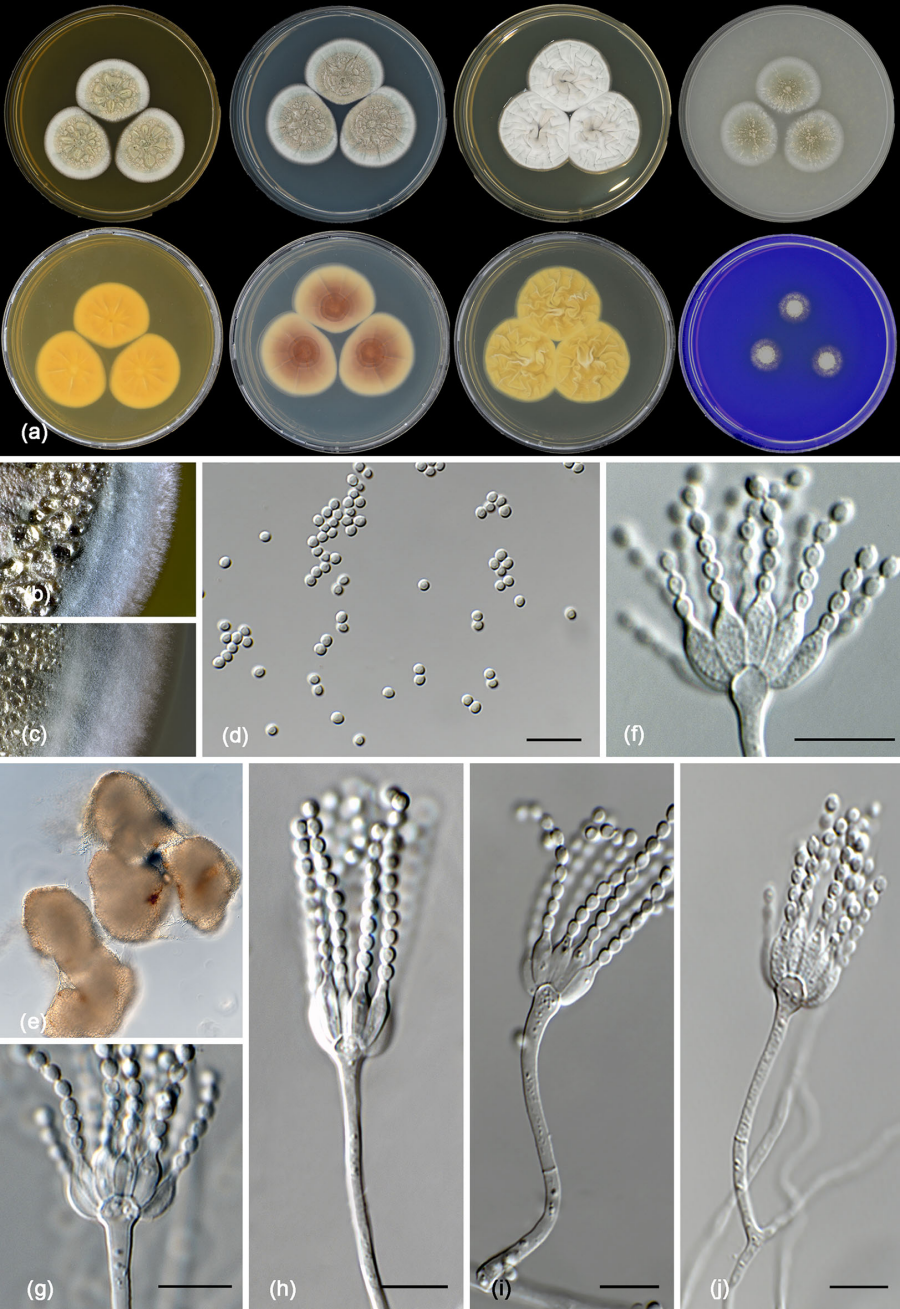
Morphological characters of *Penicillium mellis* CBS 142499. **a** Colonies from left to right (top row) MEA, CYA, YES and OA; (bottom row) CYA reverse, MEA reverse, YES reverse and CREA. **b** Texture on CYA. **c** Texture on MEA. **d** Conidia. **e** Sclerotia. **f–j** Conidiophores. Scale bars 10 μm

*Penicillium mellis* R.N. Barbosa, Souza-Motta, N.T. Oliveira & Houbraken sp. nov. (Figure 10)

MycoBank: MB822211

*Etymology*: the species name refers to honey, the substrate from which the type species was isolated.

*Diagnosis*: This species is phylogenetically distinct from other sect. *Sclerotiora* members. The conidiophores are monoverticillate, stipes vesiculate, pale to brownish coloured sclerotia are produced and no acid production on CREA is observed.

*Type*: Brazil: *Pernambuco*: Recife, honey of *Melipona scutellaris*, May 2014, *R.N. Barbosa*. Holotype (slide preparation) is deposited in the URM Mycology Herbarium (Recife, Brazil): URM 90492; ex-type strain: URM 7605 = CBS 142499.

*ITS barcode*: MF278316. Alternative markers: *BenA* = LT854643; *CaM* = LT854647; *RPB2* = LT854652.

*Colony diam, 7* *days* (*in mm*): CYA 29–30; CYA15 °C 7–8; CYA30 °C 33–35; CYA37 °C 2–4; MEA 28–30; MEA 15 °C 9–10; MEA 30 °C 33–35; MEA 37 °C no growth; DG18 24–25; CYAS 26–27; OA 24–25; YES 34–36; CREA 10–11.

*Colony characters*: CYA, 25 °C, 7 days: Colony radially sulcate; margin entire, low; mycelium white sometimes inconspicuously green; colony texture velvety; sporulation absent at centre and sparse at margins; conidial colour *en masse* greyish green; exudates present as clear droplets; soluble pigment absent; reverse brown to pale. MEA, 25 °C, 7 days: Colonies low, plane; margins low, wide, entire; mycelium white; colony texture velvety; sporulation sparse, conidial colour *en masse* greyish green; sclerotia produced, inconspicuously brown, exudate present as clear droplets; soluble pigment absent; reverse cream. YES, 25 °C, 7 days: Colonies moderately deep, radially and concentrically sulcate; margins low, narrow, entire; mycelium white, inconspicuously grey; colony texture velvety; sporulation sparse to absent, conidial colour *en masse* indeterminable; exudate absent; soluble pigment absent; reverse pale yellow. DG18, 25 °C, 7 days: Colonies plane; margins low, entire; mycelium white; colony texture velvety; sporulation strong; conidial colour *en masse* greyish green; exudate absent; soluble pigment absent; reverse pale. OA, 25 °C, 7 days: Colonies flat, margins regular; mycelium white; colony texture velvety, sporulation dense, conidial colour *en masse* greyish; exudate present as clear droplets; soluble pigment absent; reverse white to pale. CYAS 25 °C, 7 days: Colonies radially and concentrically sulcate; margins low, entire; mycelium white; colony texture velvety; sporulation moderate to strong; conidial colour *en masse* greyish green; exudate absent; soluble pigment absent; reverse brownish. CREA, 25 °C, 7 days: moderate growth, no acid production.

*Micromorphology*: Conidiophores strictly monoverticillate. Stipes smooth walled, 25–40 × 2.0–3.5 μm, vesicilate 4.0–5.0 μm. Phialides 5–12 per stipe, ampulliform, 6.5–9.0 × 2.0–3.0 μm. Conidia smooth walled, globose to subglobose, 2.0–3.0 μm. Sclerotia present, 150–250 µm.

*Additional material examined*. Brazil, Pernambuco, Recife, Inside nest of *Melipona scutellaris*, *R.N. Barbosa*, URM 7611; RB 9; RB 85; RB 110; honey of *Melipona scutellaris*, R.N. Barbosa, RB 69.

*Notes*: *Penicillium mellis* sp. nov. is phylogenetically unique. It can be distinguished from other members in section *Sclerotiora* by its ability to produce pale to brownish coloured sclerotia on MEA, CYA and OA.

**Fig. 11:**
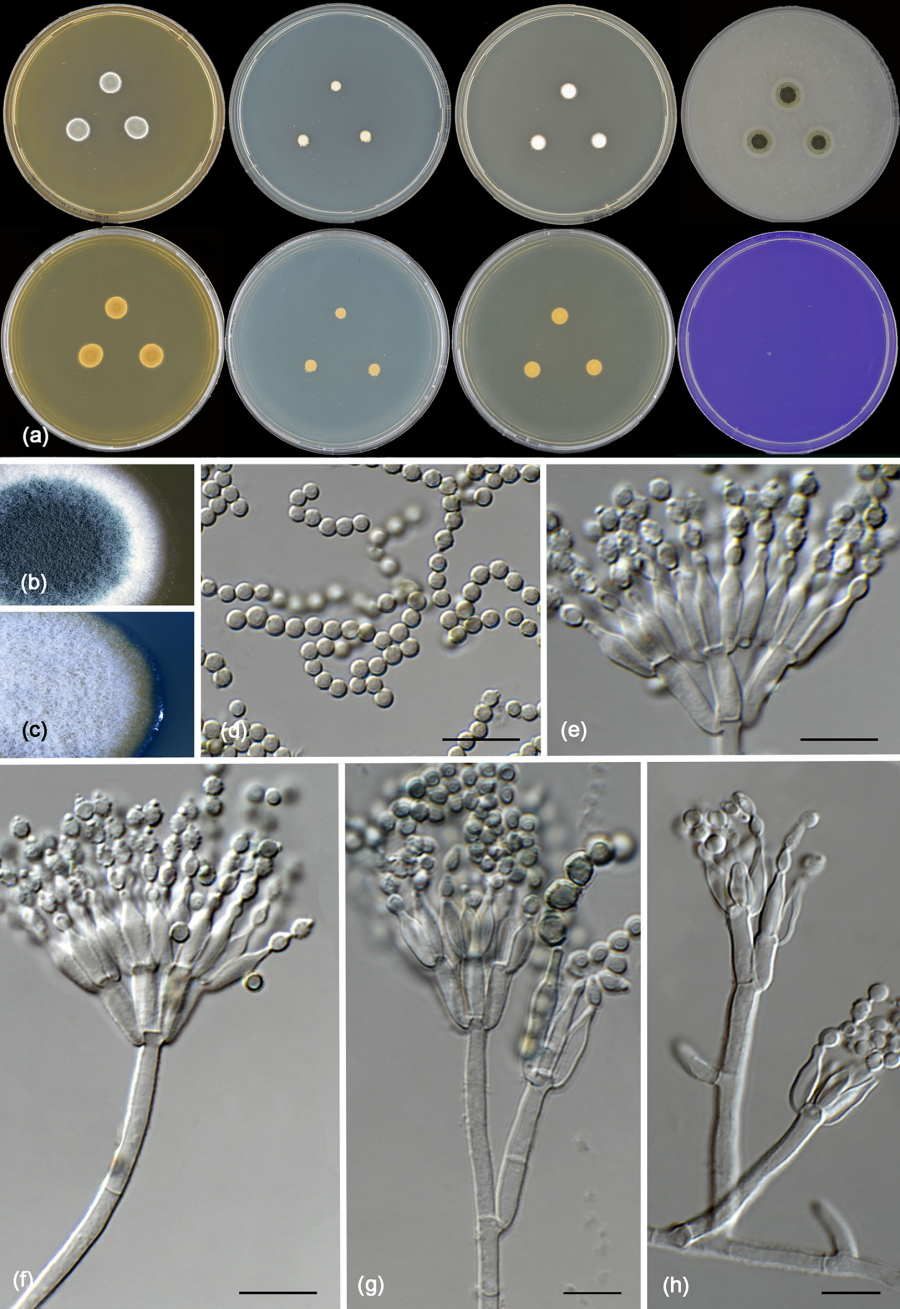
Morphological characters of *Talaromyces brasiliensis* CBS 142493. **a** Colonies from left to right (top row) MEA, CYA, YES and OA; (bottom row) CYA reverse, MEA reverse, YES reverse and CREA. **b** Texture on CYA. **c** Texture on MEA. **d** Conidia. **e–h** Conidiophores. Scale bars 10 μm

*Talaromyces brasiliensis* R.N. Barbosa, Souza-Motta, N.T. Oliveira & Houbraken sp. nov. (Figure 11)

MycoBank: MB822214

*Etymology*: Named after Brazil, the country of origin of the type strain.

*Diagnosis*: *Talaromyces brasiliensis* sp. nov. is phylogenetically unique. This species grows restricted on CYA and MEA at 25 °C and growth is absent to poor at 37 °C. The phialides of *T. brasiliensis* are ampulliform and the conidia globose and finely roughened.

*Type*: Brazil: *Pernambuco*: Recife, honey of *Melipona scutellaris*, June 2014, *R.N. Barbosa*. Holotype (slide preparation) is deposited in the URM Mycology Herbarium (Recife, Brazil): URM 90494; ex-type strains URM 7618 = CBS 142493.

*ITS barcode*: MF278323. Alternative markers: *BenA* = LT855560; *CaM* = LT855563; *RPB2* = LT855566.

*Colony diam, 7* *days* (*in mm*): CYA 5–6; CYA15 °C 3–4; CYA30 °C 5–6; CYA37 °C no growth; MEA 14–15; MEA 15 °C 6–7; MEA 30 °C 14–15; MEA 37 °C 4–5; DG18 10–11; CYAS no growth; OA 12–13; YES 6–8; CREA no growth.

*Colony characters*: CYA, 25 °C, 7 days: Colonies plane; margins entire; mycelium white; colony texture loosely floccose; sporulation poor; conidia *en masse* greyish green; exudates absent; soluble pigments absent; reverse cream to brownish. MEA, 25 °C, 7 days: Colonies plane; margins entire; mycelium white; colony texture loosely funiculose to floccose; sporulation strong; conidia *en masse* greyish; exudates absent; soluble pigments absent; reverse cream to yellow. YES, 25 °C, 7 days: Colonies loosely deep; margins entire; mycelium white; colony texture floccose; sporulation absent; conidia *en masse* indeterminable; exudates absent; soluble pigments absent; reverse cream to yellow. DG18, 25 °C, 7 days: Colonies raised at centre; margins entire, deep; mycelium white, occasionally inconspicuously grey; colony texture floccose; sporulation poor at centre, conidia *en masse* greyish; exudates absent; soluble pigments absent; reverse brown to pale. OA, 25 °C, 7 days: Colonies plane; margins entire; mycelium white, occasionally light yellow; colony texture velvety; sporulation strong at centre, week at margin; conidia *en masse* dull green; exudates present as small hyaline droplets; soluble pigments absent; reverse white to inconspicuously black. CREA 25 °C, 7 days: no growth.

*Micromorphology*: Conidiophores biverticillate, stipes smooth walled, 20–50 × 2.5–4 μm. Metulae 5–6, 8–11 × 2.5–3.5 μm. Phialides 3–4 per stipe, ampulliform tapering to very fine necks, 7–11 (–14) × 2.0–3 μm; conidia globose, finely roughened, 2–3 μm. Ascomata not observed.

*Additional material examined*. Brazil, Pernambuco, Recife, Inside nest of *Melipona scutellaris*, *R.N. Barbosa*, URM 7619; URM 7620.

*Notes*: Section *Trachyspermi* comprise species that normally grow slowly on CYA and slightly faster on MEA. *Talaromyces brasiliensis* sp. nov. also grows restricted on CYA (5–6 mm) and better on MEA (14–15 mm), confirming the phylogenetic results. *Talaromyces brasiliensis* sp. nov. is phylogenetically distinct (Fig. 6).

**Fig. 12:**
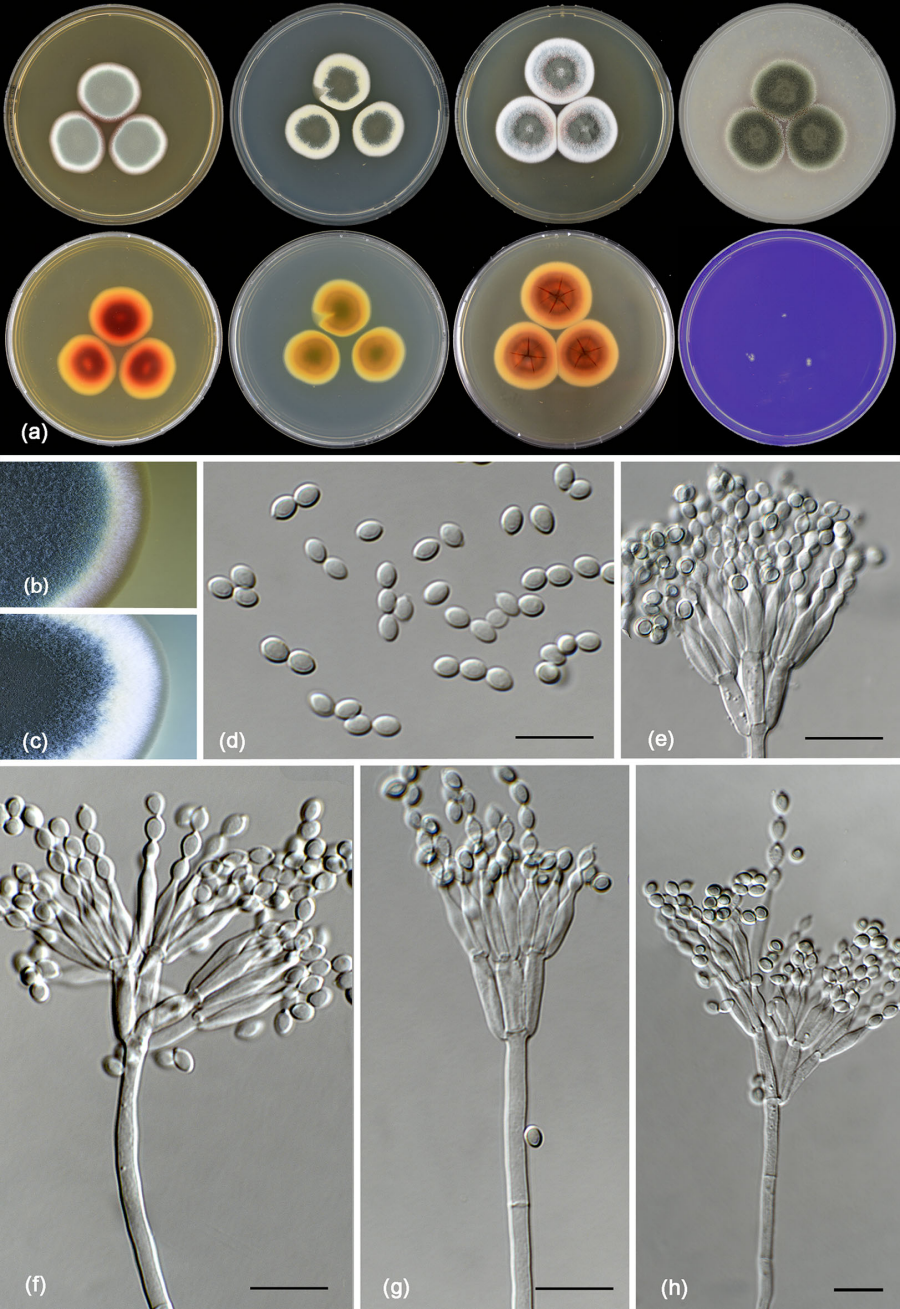
Morphological characters of *Talaromyces mycothecae* CBS 142494. **a** Colonies from left to right (top row) MEA, CYA, YES and OA; (bottom row) CYA reverse, MEA reverse, YES reverse and CREA. **b** Texture on CYA. **c** Texture on MEA. **d** Conidia. **e–h** Conidiophores. Scale bars 10 μm

*Talaromyces mycothecae* R.N. Barbosa, Souza-Motta, N.T. Oliveira & Houbraken sp. nov. (Figure 12)

MycoBank: MB822215

*Etymology*: In honour of Micoteca URM (URM, University Recife Mycology), an important Latin-American Fungal Culture Collection founded by mycologist Augusto Chaves Batista.

*Diagnosis*: The reverse colour on MEA and OA is wine red. The species produces red coloured exudate droplets on YES and no acid compounds are produced on CREA. Furthermore, *T. mycothecae* sp. nov. grows well on CYA 37 °C and produces smooth walled, fusiform to ellipsoidal shaped conidia.

*Type*: Brazil: *Pernambuco*: Recife, inside nests of *Melipona scutellaris*, Feb 2014, *R.N. Barbosa*. Holotype (slide preparation) is deposited in the URM Mycology Herbarium (Recife, Brazil): URM 90495; ex-type strains URM 7622 = CBS 142494.

*ITS barcode*: MF278326. Alternative markers: *BenA* = LT855561; *CaM* = LT855564; *RPB2* = LT855567.

*Colony diam, 7* *days* (*in mm*): CYA 20–23; CYA15 °C 2–5; CYA 30 °C 28–30; CYA 37 °C 18–20; MEA 29–30; MEA 15 °C 3–6; MEA 30 °C 38–40; MEA 37 °C 20–22; DG18 10–12; CYAS no growth; OA 24–25; YES 25–26; CREA 4–5.

*Colony characters*: CYA, 25 °C, 7 days: Colonies plane, margins entire; mycelium white occasionally inconspicuously yellow; colony texture velvety to floccose; sporulation strong, conidia *en masse* greyish to dull green; exudates present as small clear droplets; soluble pigments absent; reverse yellow amber to dark brown at centre. MEA, 25 °C, 7 days: Colonies plane; margin entire, mycelium white; colony texture velvety; sporulation strong; conidia *en masse* greyish; exudates absent; soluble pigments absent; reverse yellow amber to wine-reddish. YES, 25 °C, 7 days: Colonies crateriform; margins entire; mycelium white; colony texture floccose; sporulation strong; conidia *en masse* greyish to dull green; exudates present as small red droplets; soluble pigments absent; reverse red near margins to wine-reddish in centre. DG18, 25 °C, 7 days: Colonies plane; margins entire; mycelium white; colony texture floccose; sporulation sparse; conidia *en masse* green; exudates present as small red droplets; soluble pigments absent; reverse cream at margins to reddish at centre. OA, 25 °C, 7 days: Colonies plane; margins low; mycelium white occasionally inconspicuously greenish; colony texture velvety to granular; sporulation abundant, conidia *en masse* dull green; exudates present as small clear droplets; soluble pigments absent; reverse reddish. CREA 25 °C, 7 days: Very weak growth, acid production absent.

*Micromorphology*: Conidiophores biverticillate; stipes smooth, 55–105 × 2–3 μm; metulae 3–4, 11.5–15.5 × 2.5–4 μm. Phialides 3–5 per stipe, acerose, 9.5–12.5 × 2.5–3.5 μm. Conidia smooth, fusiform to ellipsoidal, 2.5–4 × 3–3.5 μm. Ascomata not observed.

*Additional material examined*. Brazil, Pernambuco, Recife, isolated from inside nest of *Melipona scutellaris*, *R.N. Barbosa*, URM 7623; RB 95; RB 171.

*Notes*: Altough the relationship if *Talaromyces mycothecae* sp. nov. is difficult to determine, the species seems to be phylogenetically most closely related to *T. neofusisporus*, *T. stollii*, *T. amestolkiae* and *T. ruber*. *Talaromyces neofusisporus* produces synnemata on CYA and YES, and grows poorly at 37 °C (2–3 mm, CYA, 7 days) (Wang et al. 2016). In contrast, no synnemata and good growth at 37 °C (18–20 mm, CYA, 7 days) is observed for *T. mycothecae*. Yilmaz et al. (2012) used various characters, such as the ability to grow at 37 °C, the colony texture on MEA and CYA and the production of acid compounds on CREA to differentiate *T. amestolkiae*, *T. ruber* and *T. stollii*. No acid is produced on CREA by *T. mycothecae* and this is shared with *T. ruber* (*T. amestolkiae* and *T. stollii* are poor acid producers). *Talaromyces mycothecae* sp. nov. attains a diameter of 18–20 mm after 7 days on CYA at 37 °C and this is faster than *T. amestolkiae* (8–15 mm) and *T. ruber* (14–18 mm), but slower than *T. stollii* (25–35 mm) (Yilmaz et al. 2014). Based on the data above, *T. mycothecae* phenotypically resembles *T. ruber*. The characteristic yellow and red pigmented mycelium on YES of *T. ruber* is not observed in the *T. mycothecae* sp. nov. cultures.

**Fig. 13:**
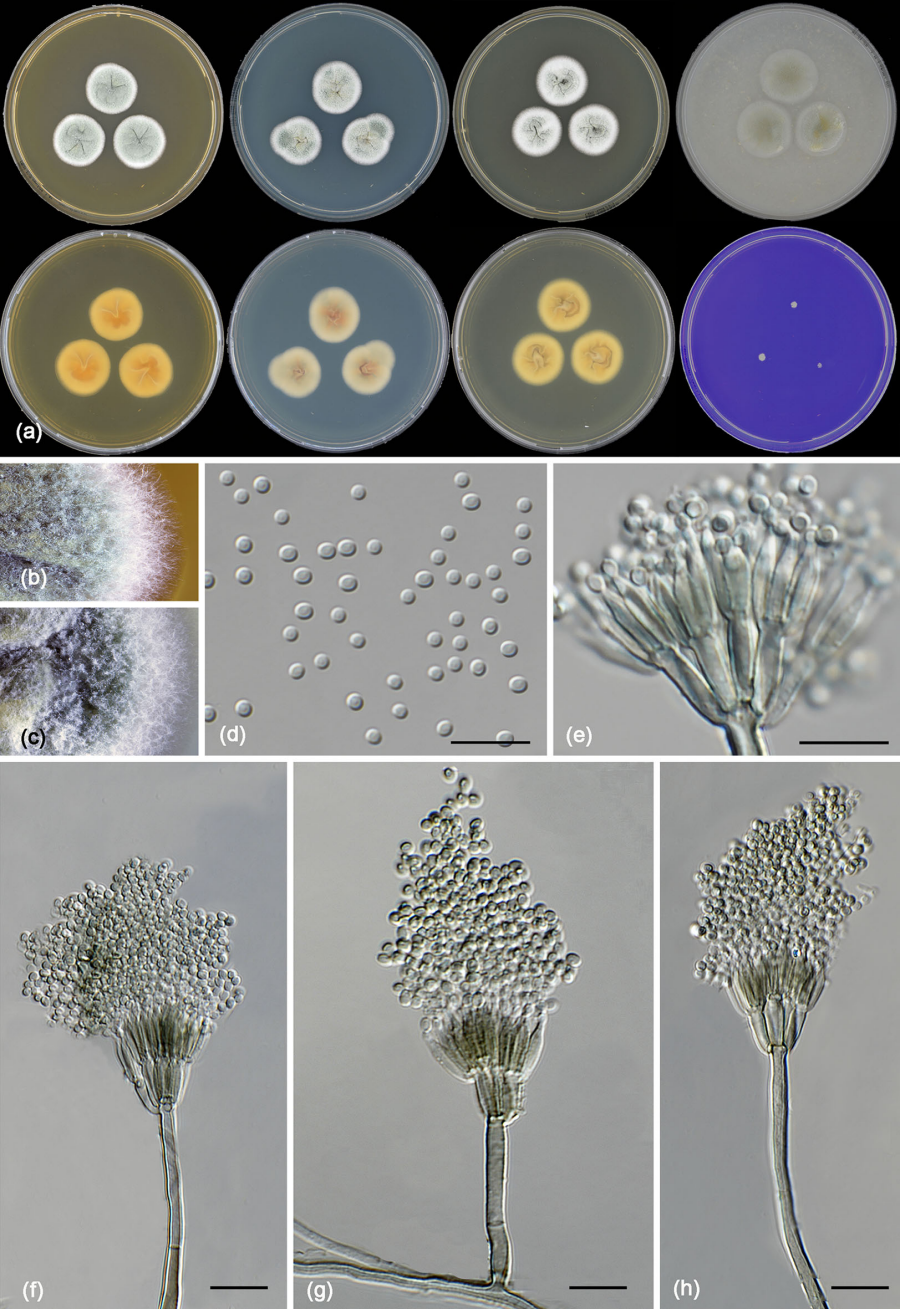
Morphological characters of *Talaromyces pigmentosus* CBS 142805. **a** Colonies from left to right (top row) MEA, CYA, YES and OA; (bottom row) CYA reverse, MEA reverse, YES reverse and CREA. **b** Texture on CYA. **c** Texture on MEA. **d** Conidia. **e–h** Conidiophores. Scale bars 10 μm

*Talaromyces pigmentosus* R.N. Barbosa, Souza-Motta, N.T. Oliveira & Houbraken sp. nov. (Figure 13)

MycoBank: MB822216

*Etymology*: Referring to the brownish green pigmented conidiophores of the species.

*Diagnosis*: *Talaromyces pigmentosus* sp. nov. is phylogenetically unique. This species produces pigmented conidiophores and grows well on CYA and MEA at 37 °C. No sexual state is observed and the species has a cream reverse on MEA and cream (margins) to brownish (centre) reverse on CYA.

*Type*: Brazil: *Pernambuco*: Recife, inside nests of *Melipona scutellaris*, June 2014, *R.N. Barbosa*. Holotype (slide preparation) is deposited in the URM Mycology Herbarium (Recife, Brazil): URM 90496; ex-type strains URM 7624 = CBS 142805.

*ITS barcode*: MF278330. Alternative markers: *BenA* = LT855562; *CaM* = LT855565; *RPB2* = LT855568.

*Colony diam, 7* *days* (*in mm*): CYA 23–24; CYA 15 °C 5–7; CYA 30 °C 34–35; CYA 37 °C 35–36; MEA 23–24; MEA 15 °C 6–8; MEA 30 °C 34–35; MEA 37 °C 33–34; DG18 7–9; CYAS 2–3; OA 24–25; YES 23–24; CREA 2–4.

*Colony characters*: CYA, 25 °C, 7 days: Colonies moderately deep; margins entire; mycelium white sometimes inconspicuously green; colony texture velvety; sporulation absent; conidial colour *en masse* cannot be determinate; exudates absent; soluble pigments absent; reverse white to cream at margins to brownish in centre. MEA, 25 °C, 7 days: Colonies moderately deep, sunken at centre; margin entire; mycelium white; colony texture velvety; sporulation sparse; conidia *en masse* greyish; exudates present as small hyaline droplets; soluble pigments absent; reverse cream. YES, 25 °C, 7 days: Colonies moderately deep, sunken, raised at centre; margins entire; mycelium white; colony texture floccose; sporulation sparse to absent, conidia *en masse* greyish; soluble pigments absent; exudates absent; reverse cream to yellow. DG18, 25 °C, 7 days: Colonies raised at centre; margins low, plane; mycelium white; colony texture floccose; sporulation absent, conidia *en masse* indeterminable; exudates absent; soluble pigments absent; reverse brown at centre, light cream to white at margin. OA, 25 °C, 7 days: Colonies low, plane; margins low, plane; mycelium white; colony texture velvety; sporulation absent; conidia *en masse* indeterminable; exudates absent; soluble pigments absent; reverse light cream. CYAS, 25 °C, 7 days: Colonies low, plane; margins low, plane; mycelium white; colony texture velvety; sporulation absent; conidia *en masse* indeterminable; exudates absent; soluble pigments absent; reverse white. CREA 25 °C, 7 days: Very weak growth, acid production absent.

*Micromorphology*: Conidiophores biverticillate, brownish green pigmented. Stipes smooth walled, 17–65 × 2–4 μm. Metulae 3–4, divergent, 7–11 × 2–2.5 μm. Phialides, 3–6 per stipe, acerose, 7–11 × 2–3 μm; conidia smooth walled, globose to subglobose, 2.0 × 3.0 μm. Ascomata not observed.

*Additional material examined*. Brazil, Pernambuco, Recife, from bee pollen of *Melipona scutellaris*, *R.N. Barbosa*, URM 7625; RB 96, RB 171; from inside nest of *Melipona scutellaris* RB 30; RB 100.

*Notes*: *Talaromyces pigmentosus* sp. nov. is phylogenetically closely related to *T. helicus*, *T. boninensis* and *T. reverso*-*olivaceus* and shares the ability to grow well on CYA incubated at 37 °C (10–34 mm, 7 days). *Talaromyces boninensis* and *T. helicus* produce a sexual state and this is not observed *T. reverso*-*olivaceus* and *T. pigmentosus* sp. nov.. The new species can also be differentiated from *T. reverso*-*olivaceus* by its ability to produce brownish green pigmented stipes. The production of pigmented stipes is also shared with the phylogenetically more distant species *T. varians* (also in sect. *Helici*) and *T. ptychoconidium* (sect. *Purpurei*) (Yilmaz et al. 2014).

## Discussion

*Penicillium* and *Talaromyces* species are well-known cosmopolitan filamentous fungi that play various roles in natural ecosystems, agriculture and biotechnology. Both genera have a sectional infrageneric classification system. Currently, 26 sections are accepted in *Penicillium* (Houbraken and Samson 2011; Houbraken et al. 2016b) and seven in *Talaromyces* (Yilmaz et al. 2014). A current monograph on *Penicillium* is lacking, but many *Penicillium* sections are studied using a polyphasic approach or multigene phylogenies (e.g. Houbraken et al. 2014; Peterson et al. 2015; Visagie et al. 2015; Houbraken et al. 2016b) and all *Talaromyces* sections were treated in detail in the monograph of Yilmaz et al. (2014). ITS and *BenA* sequences are proposed identification markers for *Penicillium* and *Talaromyces* and often are generated in taxonomic studies. In many of those studies it is shown that ITS sequencing is insufficient for *Penicillium* identifications as closely related species often share similar or identical sequences. On the other hand, this locus works relatively well to assign species to sections. Besides ITS sequences, we also generated *BenA* sequences of all isolated *Penicillium* and *Talaromyces* species. The *BenA* sequences are used for identification of *Penicillium* and *Talaromyces* species, as this is the recommended identification marker (Visagie et al. 2014; Yilmaz et al. 2014). With exception of the new species, all other isolates obtained during our survey could be reliably identified using *BenA* sequences.

Among the Penicillia, section *Sclerotiora* (46%) isolates were most frequently detected during this study. *Penicillium brocae* (27%) was predominantly present among the isolates belonging to section *Sclerotiora*, followed by *P. mellis* sp. nov. (7%), *P. sclerotiorum* (5%)*, P. mallochii* (4%), *P. sanshaense* (1%), *P. fernandesiae* sp. nov. (1%) and *P. meliponae* sp. nov. (1%). Section *Sclerotoria* species generally produce monoverticillate conidiophores and exceptions are the biverticillate conidiophores observed in *P. choerospondiatis*, *P. herquei*, *P. malachiteum, P. sanshaense* and *P. verrucisporum*. They also have bright yellow or orange pigments, which may occur in the mycelium, sclerotia, ascocarps, soluble pigments and/or colony reverse pigmentation (Houbraken and Samson 2011; Rivera and Seifert 2011; Visagie et al. 2013). The species isolated during our survey also produced monoverticillate conidiophores, bright coloured colonies, sclerotia and/or mycelium, and none of the strains produced a sexual state. Interestingly, *P*. *brocae* was the most predominant *Penicillium* present in our study. This species was originally described from coffee berry borers (galleries, cuticle, feces and guts) in Mexico (Peterson et al. 2003), and more recently detected in faeces of another, unrelated beetle, *Eufallia* sp. (Wang and Chan 2015). Along with the description of *P. brocae*, Peterson et al. (2003) suggested that this species produces exogenous sterols necessary for the coffee berry borer’s development and thus is mutualistically associated with the insect. Similar to other insects, bees are unable to synthesize sterols and, thus, exogenous sterol is required (Ferreira-Caliman et al. 2012). A recent study showed that essential steroid precursors, needed for the development of *Scaptotrigona depilis* bees, are proved by a *Zygosaccharomyces* species (Paludo et al. 2018). Whether a symbiosis exists between steroids produced by *Penicillium* and *Talaromyces* species and *Melipona scutellaris* bees needs to be further investigated. Also *P. mallochii* and *P. guanacastense*, two species related to *P. brocae*, are associated with guts and faeces of leaf-eating caterpillars (Rivera et al. 2012) suggesting an association of other section *Sclerotiora* members with insects as well.

Isolates belonging to section *Citrina* made up for 30% of all Penicillia. *Penicillium citrinum* was most frequently detected (20%), followed by *P. steckii* (5%), *P. shaerii* (4%), *P. paxilli* (2%), and *P. sumatrense* (1%). These species are characterised by symmetrically biverticillate conidiophores, flask shaped phialides, small-sized conidia, and some species like *P. shaerii* produce greyish brown cleistothecia. These species are common in soils and have a preference for (sub)tropical soils. It’s unknown whether these species are saprotrophs or if they are associated with stingless bees. Section *Citrina* species produce various bioactive extrolites (mycotoxins, antibiotics) such as citrinin, curvularin, paspaline, paspalinine and paxillin (Houbraken et al. 2011a). The presence of these extrolites in honey and pollen samples wasn’t subject of this study; however, if present, they can affect the quality of the honey and pollen. How insects cope with mycotoxins has rarely been investigated (Gliński and Jarosz 2000; Traniello et al. 2001; Keller et al. 2014).

Another group of isolates obtained in this study belong to *Lanata*-*Divaricata*, a section re-established by Houbraken and Samson (2011). Most of the section *Lanata*-*Divaricata* species grow rapidly in culture. The conidiophores of these species are often strongly divaricate and have metulae that are born terminally, subterminally and in intercalary positions. Useful characters for identification are the shape and ornamentation of the conidia, growth on CYA incubated at 37 °C, colony diameters and morphology (e.g. reverse colours on CYA, YES), and growth rate on CREA. Three *Lanata*-*Divaricata* species were isolated: *P. singorense*, *P. wotroi* and *P. echinulonalgiovense*. One isolate (RB 115) represents a novel species in sect. *Lanata*-*Divaricata*, and this species will be described elsewhere (Y-Z Diao et al., in progress). *Penicillium singorense* and *P. wotroi* are known species and the former was originally described from house dust in Thailand. Recent collections show that *P. singorense* has a worldwide distribution and this species is isolated in USA (Florida), Korea and China (J. Houbraken, pers. observations). The distribution of *P. wotroi* seems to be restricted to South-America (Brazil, Argentina) (Houbraken et al. 2011b). The name *P. echinulonalgiovense* was invalidly published because it was described without a Latin description or diagnosis (Art. 39.1.; Melbourne Code). In subsequent treatments using morphological characters, this species was placed in synonymy with *P. janthinellum* (Smith 1963) and/or *P. simplicissimum* (Pitt 1979; Stolk and Samson 1983). However, molecular data shows that this species is distinct (Houbraken et al. 2011b) and we therefore reinstate this species as distinct. A search in the DTO and CBS collection shows that this species has a worldwide distribution (J. Houbraken, unpubl. results). Section *Lanata*-*Divaricata* species are usually found in soil and (decaying) leaves (Houbraken et al. 2011b), but various other substrates are listed in literature. These species probably have a broad ecological niche. For example *P. excelsum* was isolated from bees and ants, but also from other substrates such as flowers, leaves, Brazil nut kernels and shells (Taniwaki et al. 2015).

Insects are adapted to different ecosystems and have symbiotic and/or pathogenic associations with fungi and other microorganisms (Bode 2011; Mello et al. 2016). Studies investigating the fungi associated with bees (*sensu lato*) revealed that some fungi are common saprophytes in the environment of the beehive. Bees collect plant pollen and nectar from different kinds of plants and inevitably, also fungal fragments will be introduced to the bee pollen (e.g. Eltz et al. 2002; Barbosa et al. 2017; Paludo et al. 2018). The nutritional value of a fungal spore is lower than that of pollen; however, it is speculated that fungal spores can serve as a complement to the bee diet since the availability is high and harvest is relatively easy (Oliveira and Morato 2000; Eltz et al. 2002). Only a limited number of reports about fungi associated with stingless bees are found in literature, and the reports related to *Penicillium* and *Talaromyces* are even rarer (Ferraz et al. 2008). According to Pandey et al. (1983), pollen grains can secret substances that inhibit microbial spore germination. After collection, the pollen grains are processed by bees into bee bread and this product normally has low water activity. Many different fungal species can be found in honey, but these are probably latently present and will not grow due to the low water activity of the product. The natural introduction of fungi in the bee environment most likely occurs in the period between collection of the plant pollen, formation of bee pollen and drying and storage of the pollen in the nests.

An important characteristic of *Penicillium* and *Talaromyces* species is the production of a diverse range of bioactive extrolites (Nielsen et al. 2017). The extrolite production of the species that were isolated during this study was investigated. The (combination of) detected extrolites can potentially play a role in the interaction between different organisms (Frisvad 2008). Our results demonstrate that the detected species are able to produce several extrolites, including the mycotoxin citrinin. The occurrence of mycotoxins and mycotoxigenic fungi has been recorded in bee pollen (*sensu lato*) around the world (e.g. González et al. 2005; Kačániová et al. 2011; Rodríguez-Carrasco et al. 2013). Logically, the presence of this mycotoxins (e.g. citrinin) is unwanted and can negatively affect the quality of the bee pollen for human consumption. Extrolite function depends on their ecological interaction. Insects are well adapted to feed on plants that contain a broad spectrum of (chemical) compounds (Dowd 1992). They have a long evolutionary history of interacting with fungi and it is known that some insects can use fungi as feed (Dowd 1992). Often, mycotoxins co-occur with other fungal extrolites for which no function is known. Following the analogy with the situation in higher plants, it is possible that these co-occurring fungal extrolites can synergize (or antagonize) the toxicity of co-occurring mycotoxins (Dowd 1992). In nature, fungal metabolites can provide various fitness advantages ranging from protection to competition with other microbes for niche securement (Rohlfs and Churchill 2011). *Penicillium apimei* sp. nov. and *Monascus* are both isolated from stingless bees and are both producers of compounds belonging to the geodin biosynthetic family (Barbosa et al. 2017). It is tempting to speculate that this group of metabolites might have particular function in the bee habitat. Various extrolites could not be identified in our experimental conditions and could represent novel bioactive compounds. During this study, the quality of the nests and the health of the bees was followed over a long time and no disease was observed. If any of these (novel) compounds were secreted in the bee pollen or nests, then these compounds probably didn’t have an (large) effect on the bees health.

Data on the functional relationship between fungi and stingless bees are scarce. Further studies on fungi from honey, inside nests, bee body, stored pollen and native plants are needed to understand the relationships between these organisms in tropical ecosystems, and the benefits that such fungi can possibly confer on their hosts. This study could serve as the first step for more detailed studies on ecological interactions between stingless bees, fungi and their bioactive extrolites.

## Electronic supplementary material

Below is the link to the electronic supplementary material.

Supplementary material 1 (PDF 151 kb)

Supplementary material 2 (PDF 343 kb)

Supplementary material 3 (PDF 338 kb)

Supplementary material 4 (PDF 268 kb)

Supplementary material 5 (PDF 352 kb)

Supplementary material 6 (PDF 400 kb)

Supplementary material 7 (PDF 477 kb)

Supplementary material 8 (PDF 491 kb)

Supplementary material 9 (PDF 249 kb)
