# Supplementary figures and images for: New Penicillium and Talaromyces species from honey, pollen and nests of stingless bees
Source: Antonie Van Leeuwenhoek. 2018 Apr 13;111(10):1883–912. doi: 10.1007/s10482-018-1081-1 (PMC6153986; doi:10.1007/s10482-018-1081-1)

ITS

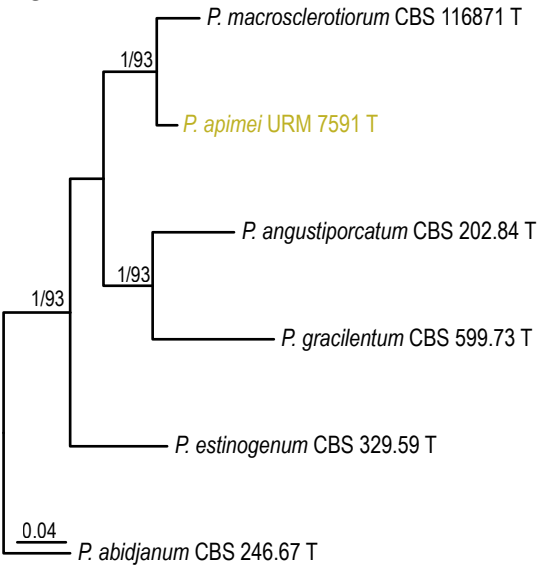

BenA

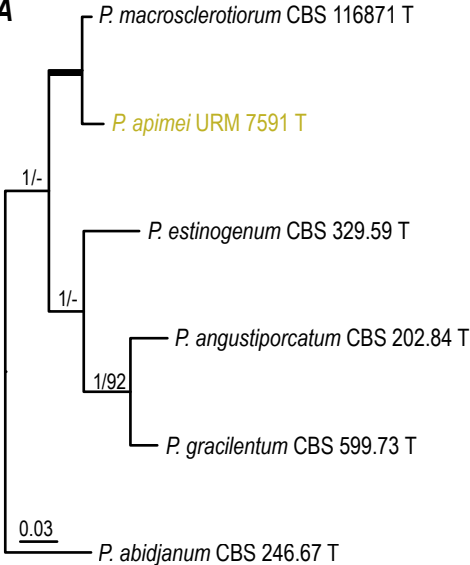

CaM

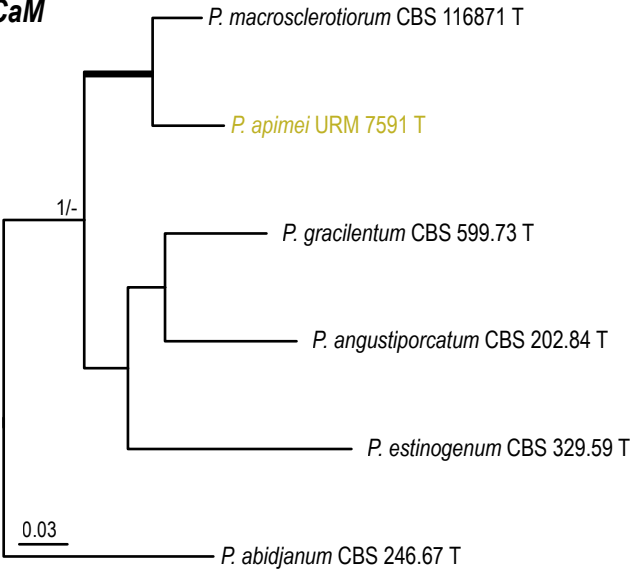

RPB2

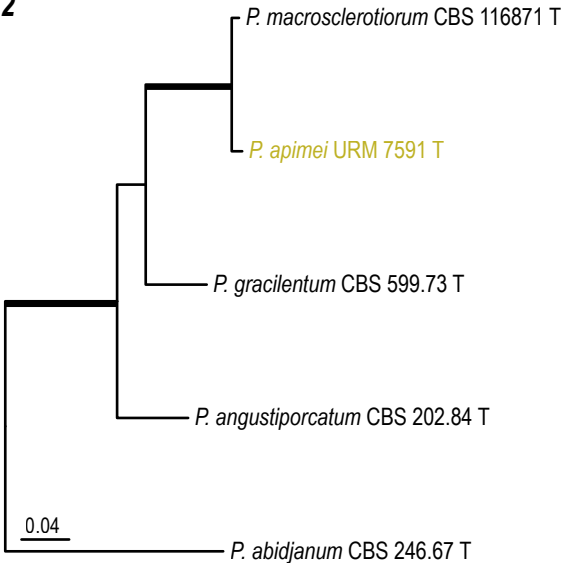

Supplement: Supplementary file 1 — Supplementary material 1 (PDF 151 kb) [file 10482_2018_1081_MOESM1_ESM.pdf]

# ITS

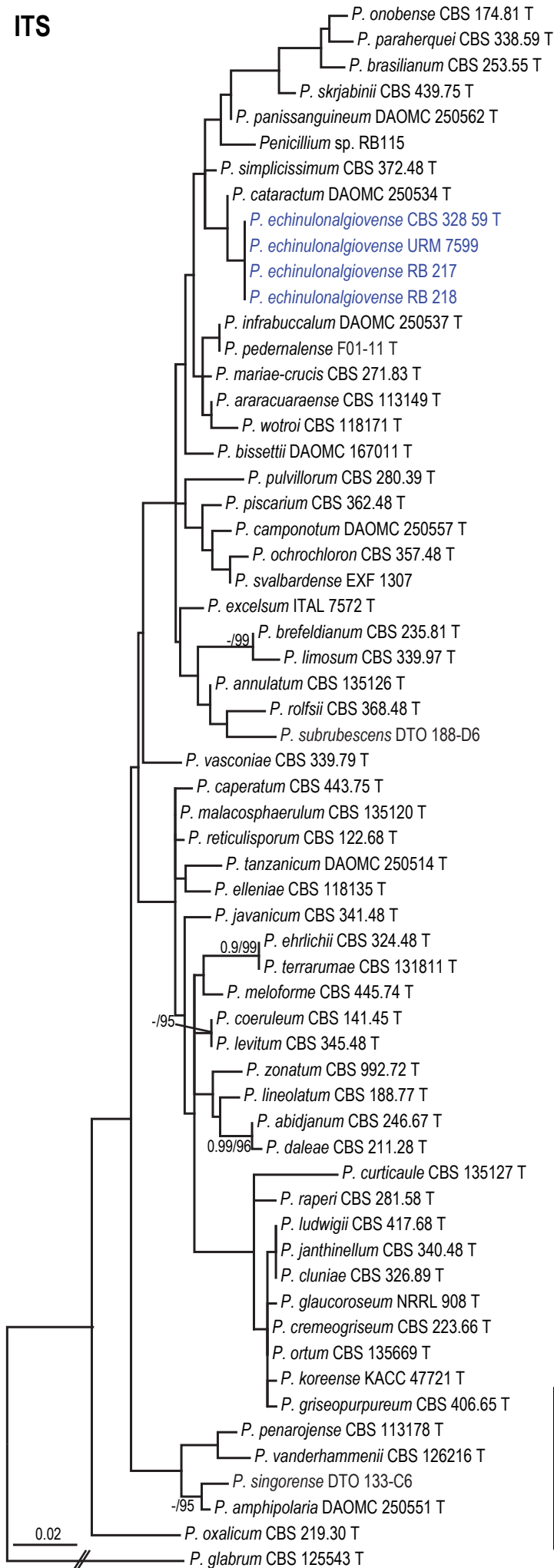

# BenA

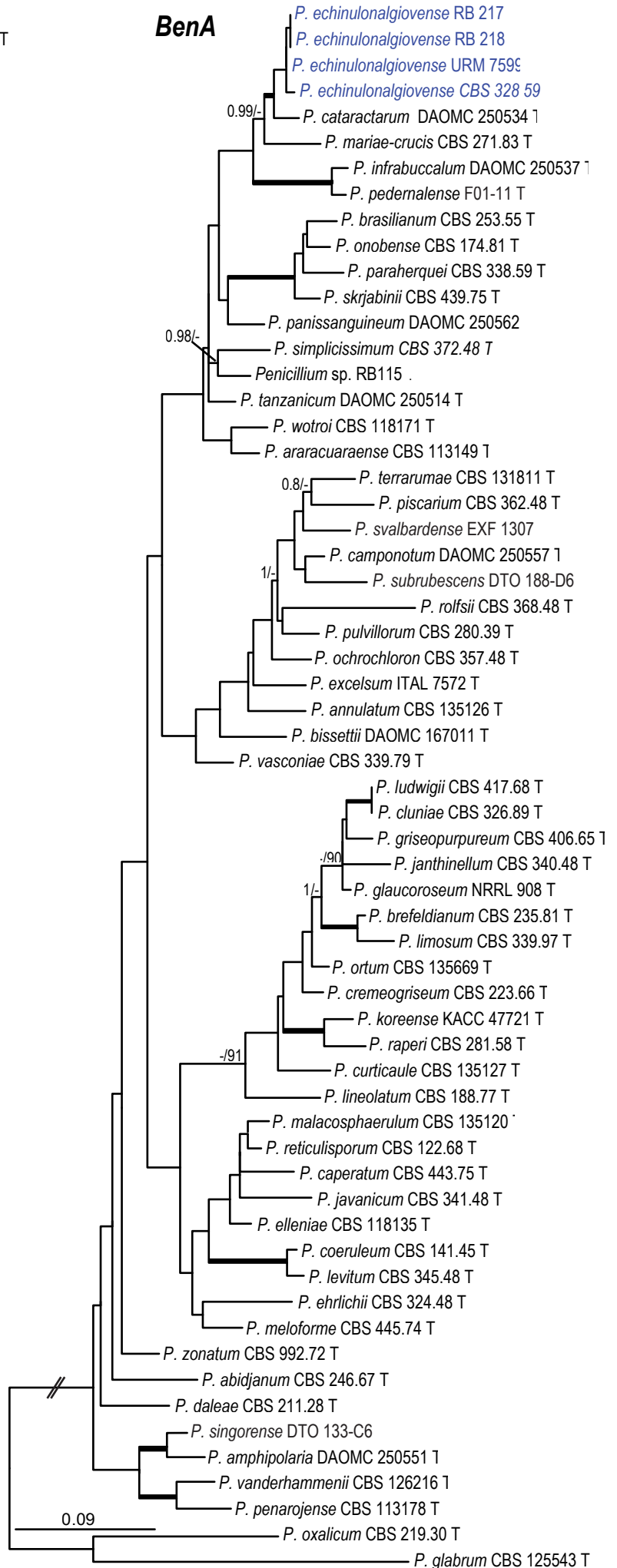

Supplement: Supplementary file 2 — Supplementary material 2 (PDF 343 kb) [file 10482_2018_1081_MOESM2_ESM.pdf]

# CaM

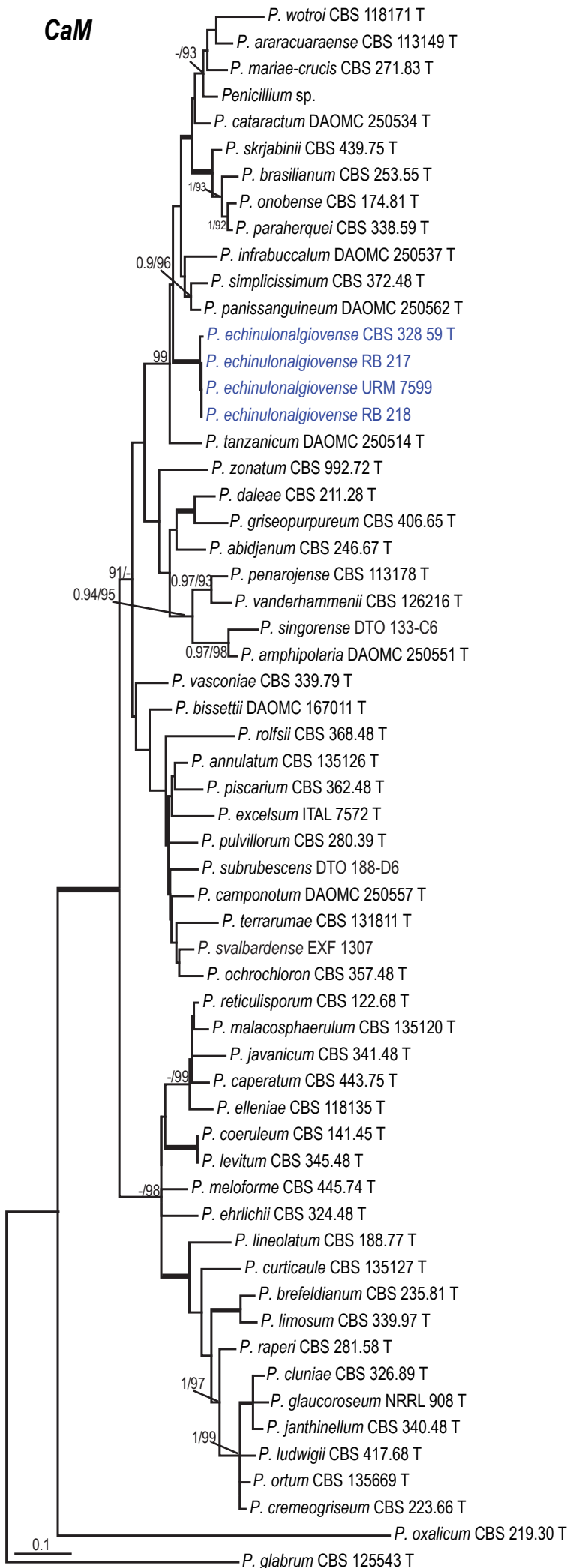

# RPB2

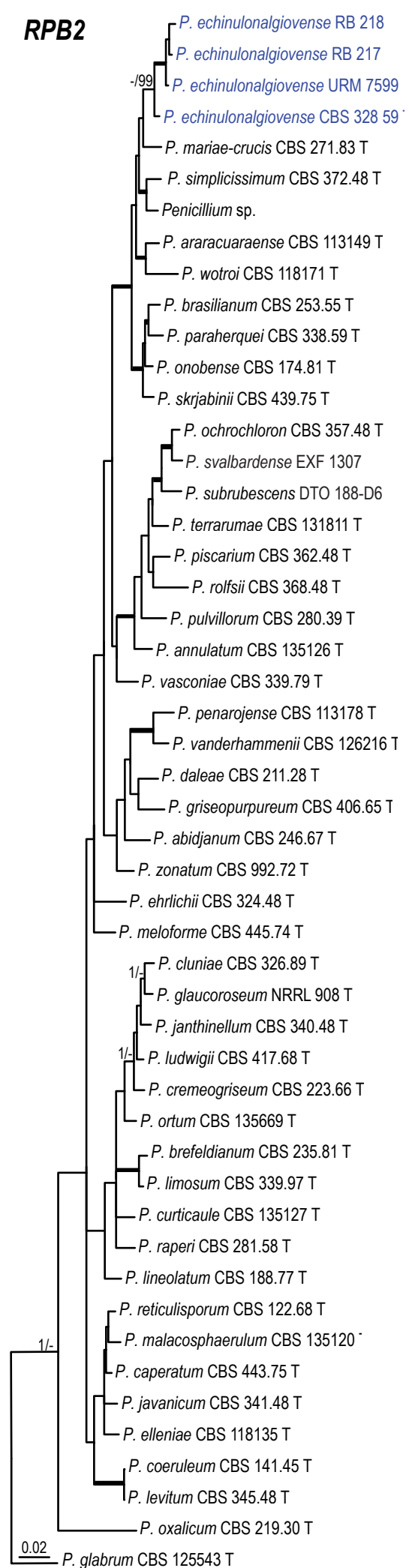

Supplement: Supplementary file 3 — Supplementary material 3 (PDF 338 kb) [file 10482_2018_1081_MOESM3_ESM.pdf]

## ITS

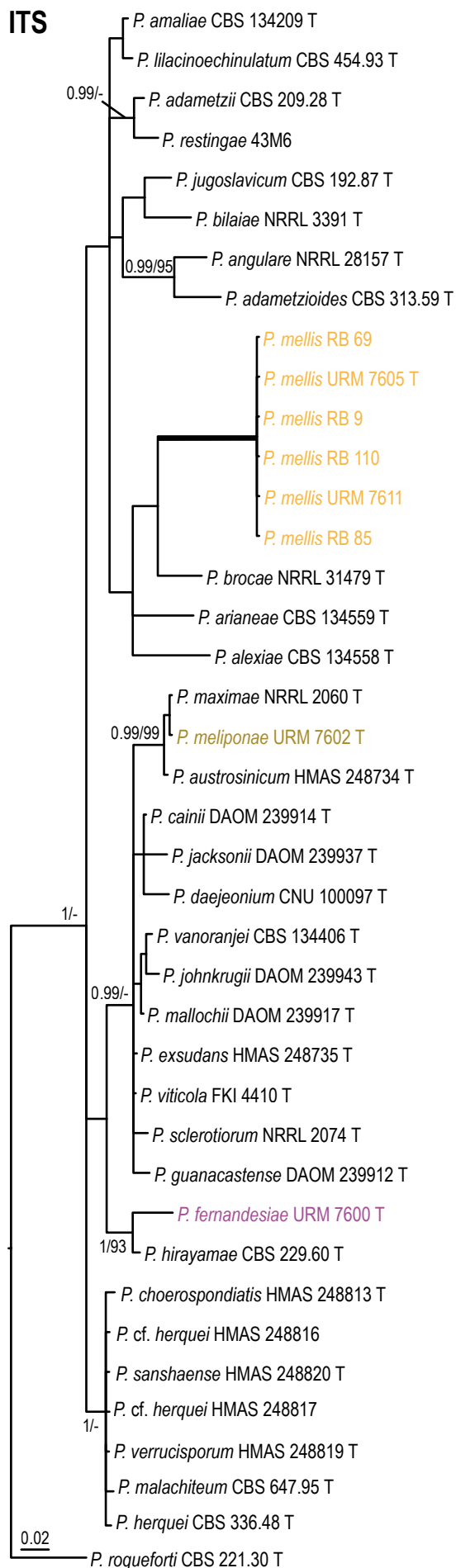

## BenA

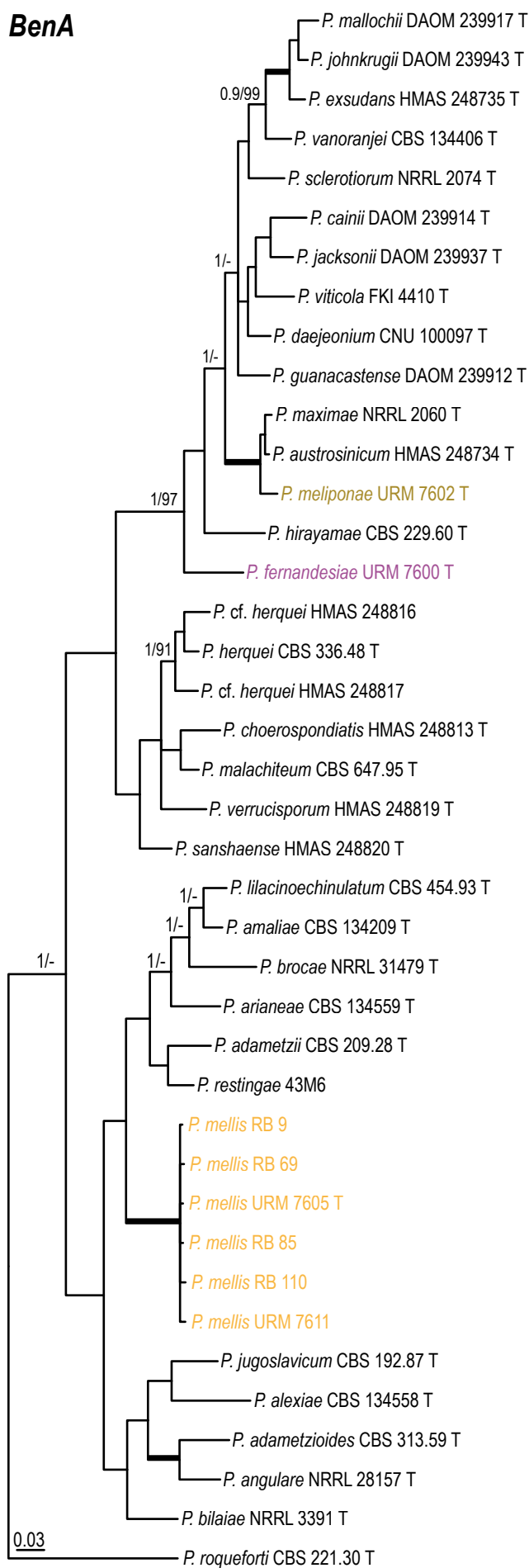

Supplement: Supplementary file 4 — Supplementary material 4 (PDF 268 kb) [file 10482_2018_1081_MOESM4_ESM.pdf]

CaM

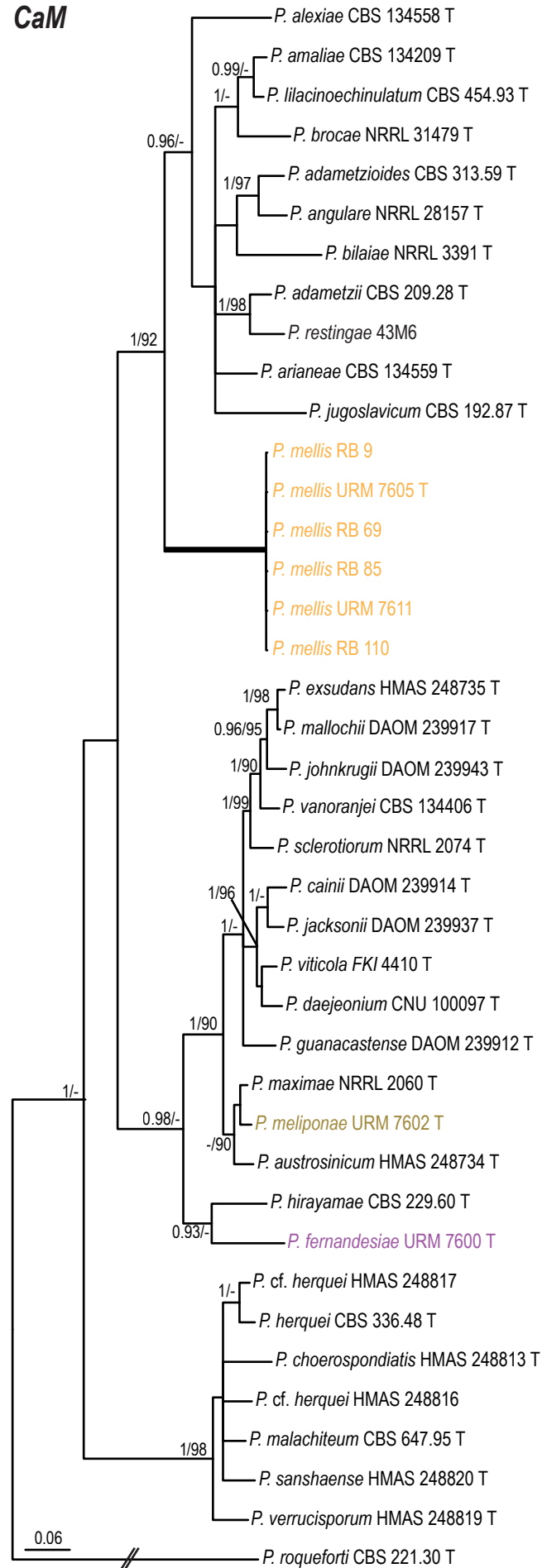

Supplement: Supplementary file 5 — Supplementary material 5 (PDF 352 kb) [file 10482_2018_1081_MOESM5_ESM.pdf]

## ITS

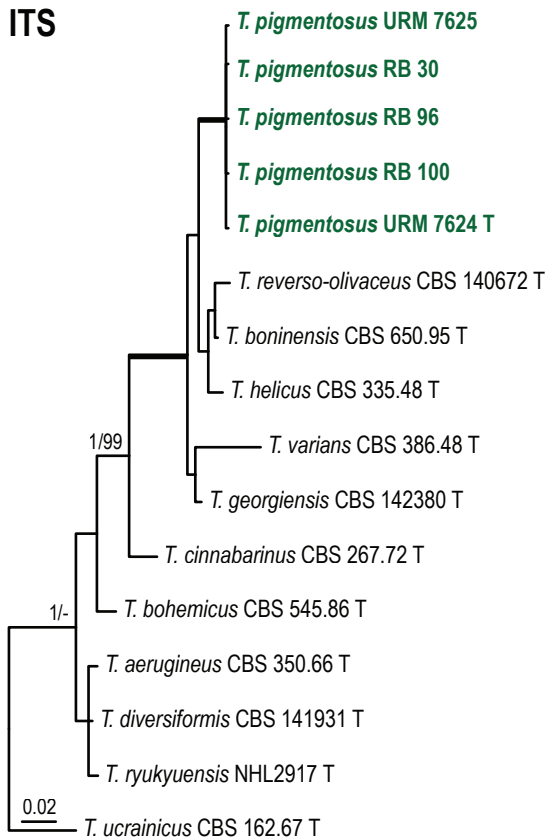

## BenA

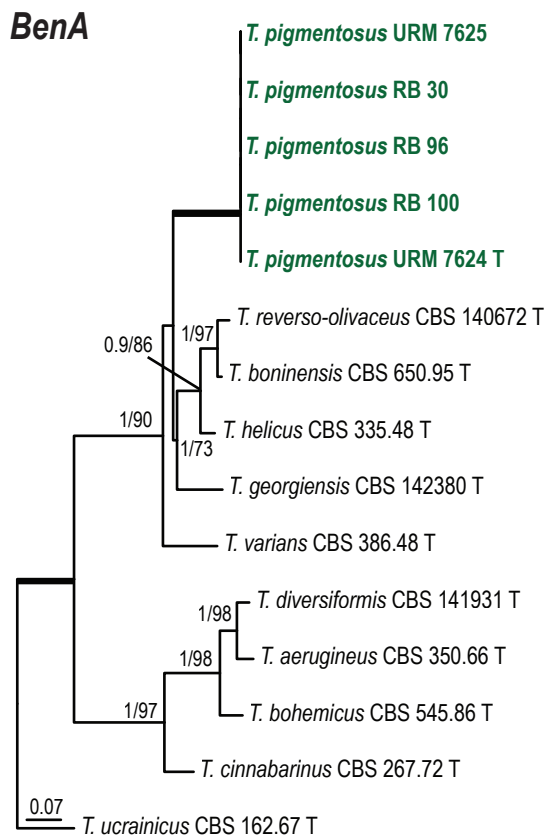

## CaM

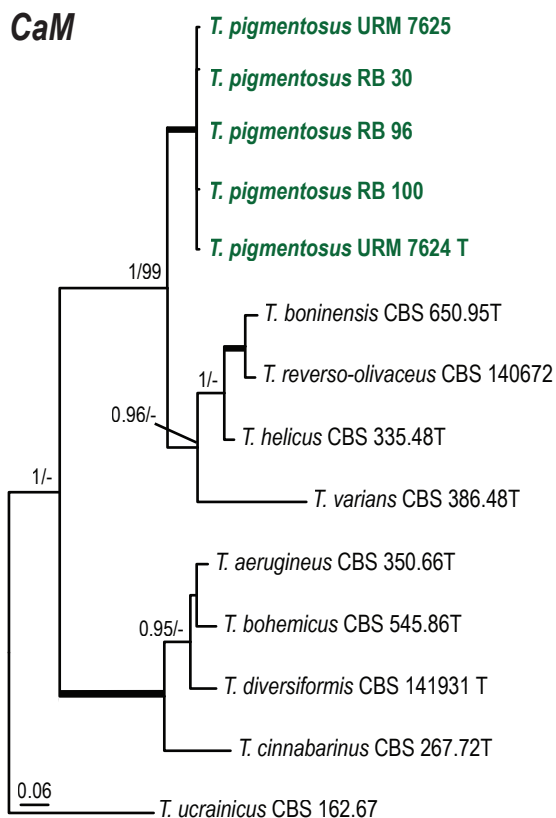

## RPB2

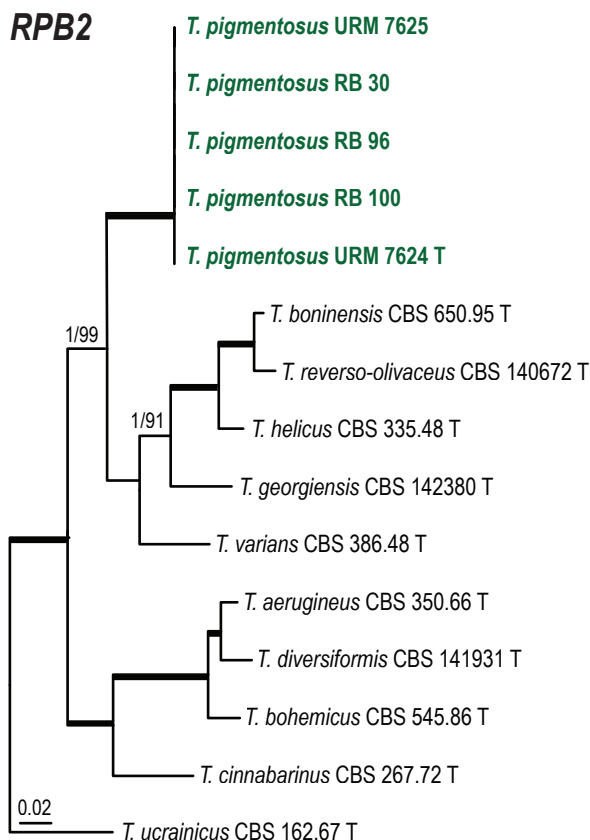

Supplement: Supplementary file 6 — Supplementary material 6 (PDF 400 kb) [file 10482_2018_1081_MOESM6_ESM.pdf]

## ITS

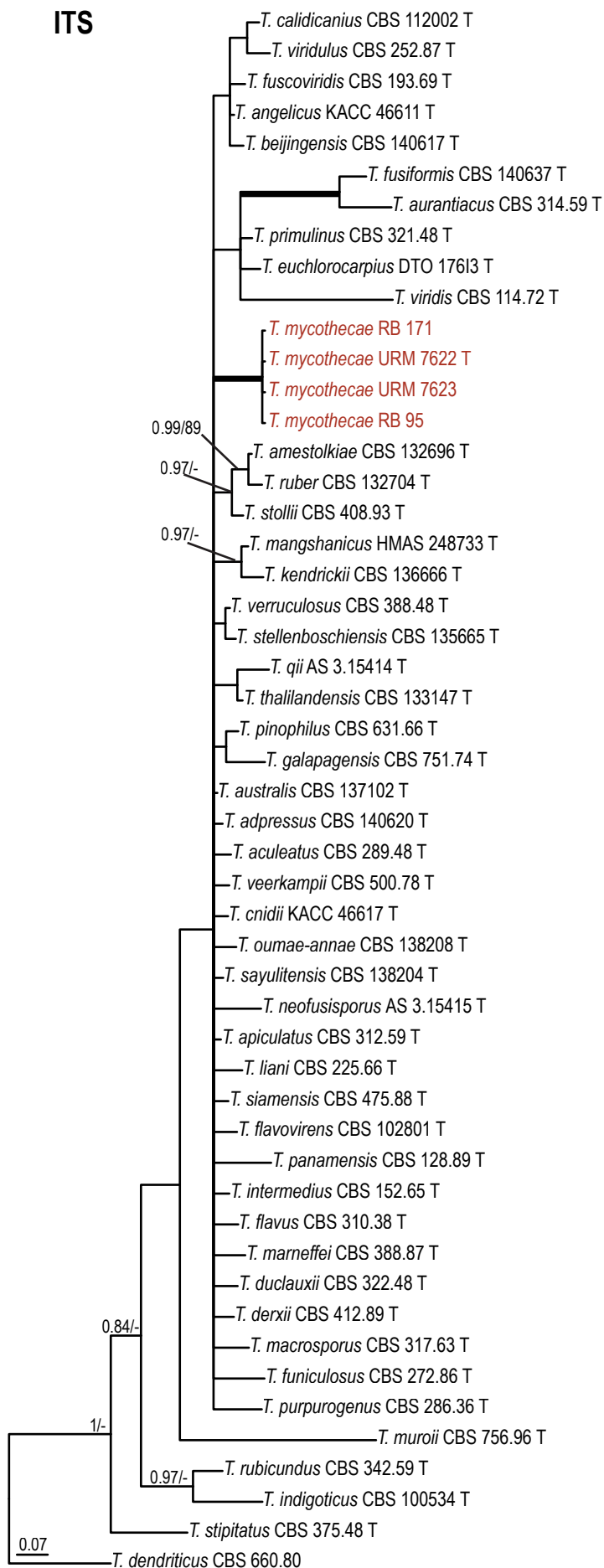

## BenA

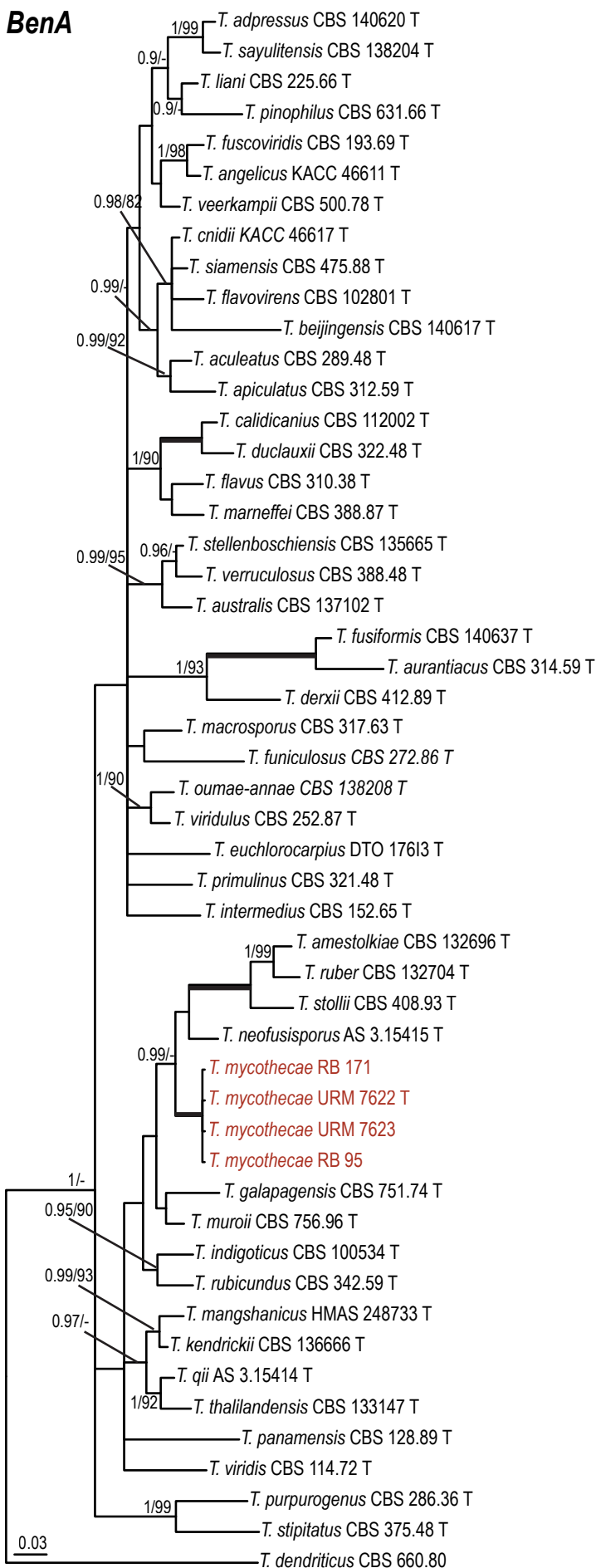

Supplement: Supplementary file 7 — Supplementary material 7 (PDF 477 kb) [file 10482_2018_1081_MOESM7_ESM.pdf]

**CaM**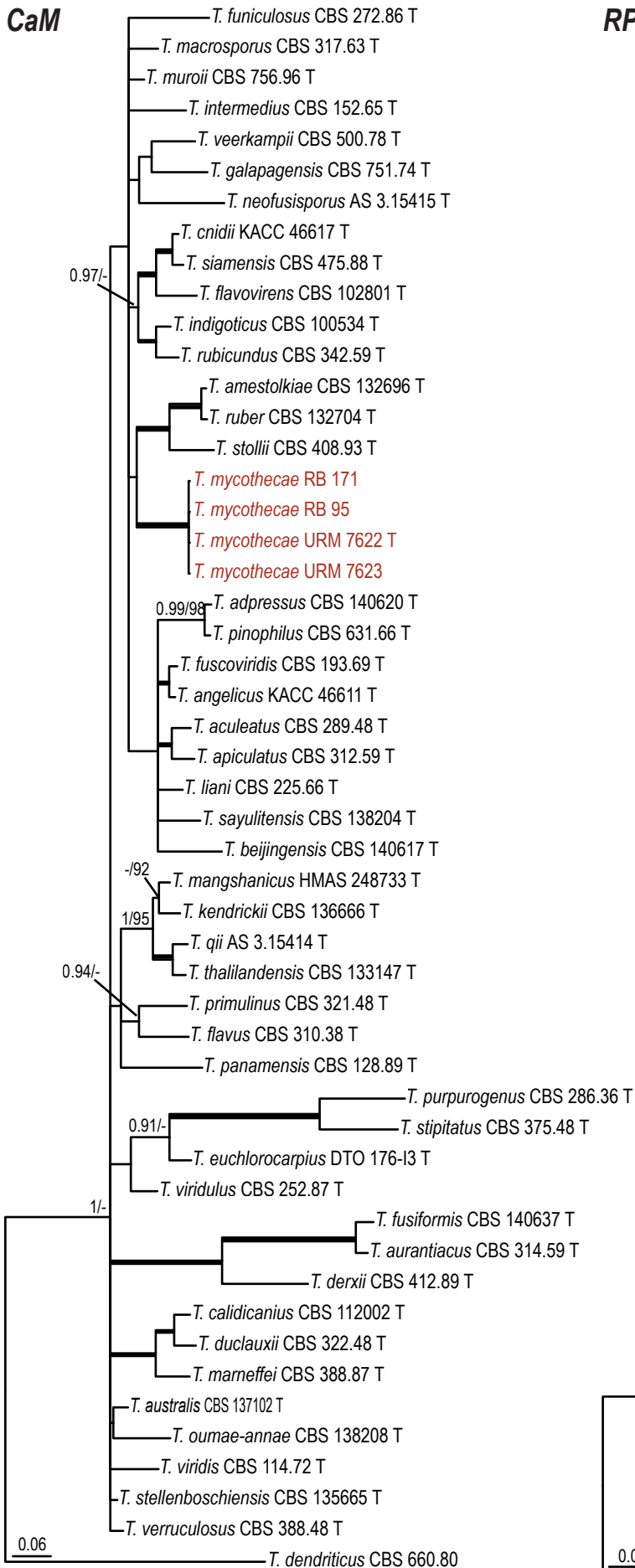**RPB2**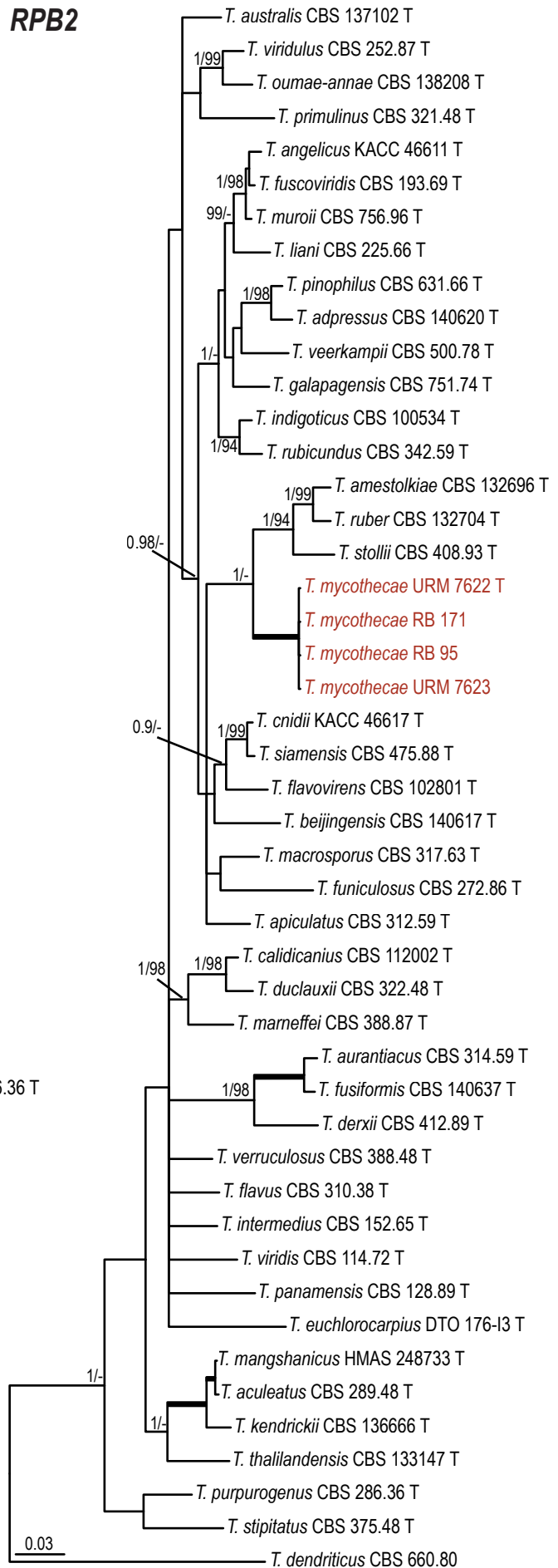

Supplement: Supplementary file 8 — Supplementary material 8 (PDF 491 kb) [file 10482_2018_1081_MOESM8_ESM.pdf]

## ITS

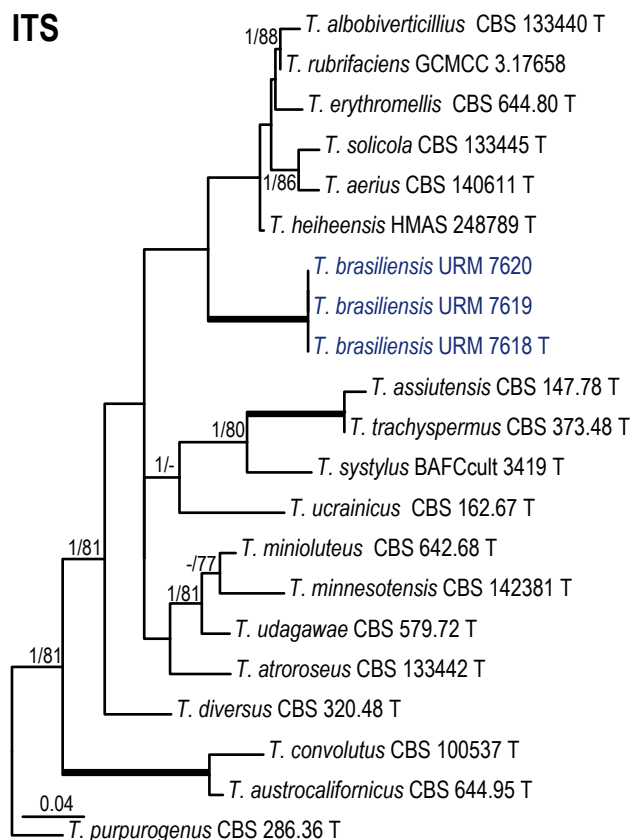

## BenA

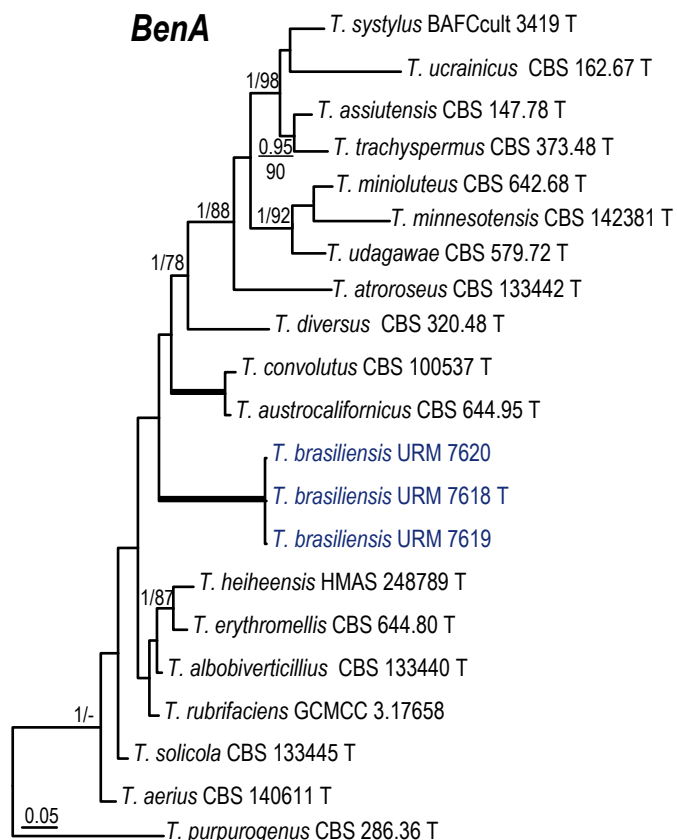

## CaM

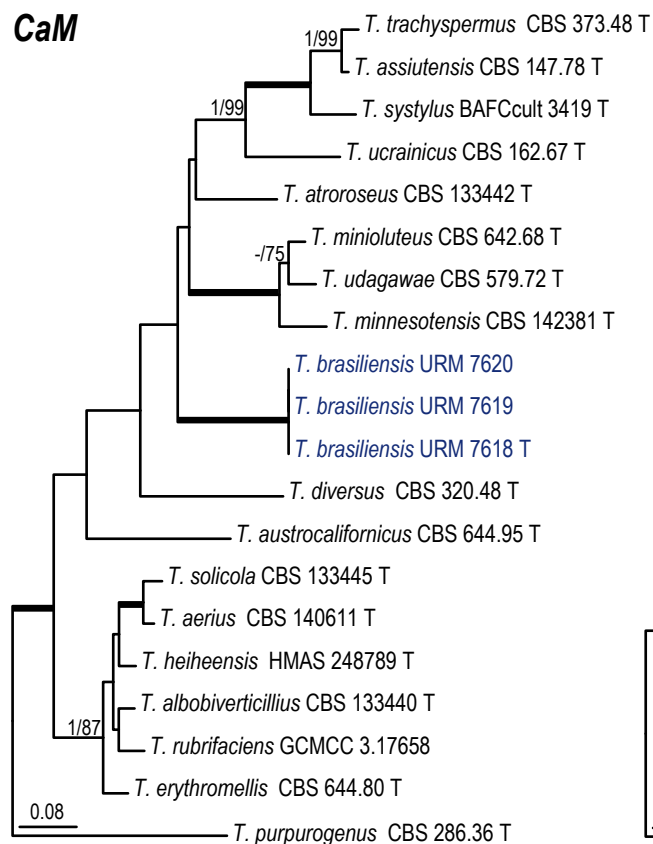

## RPB2

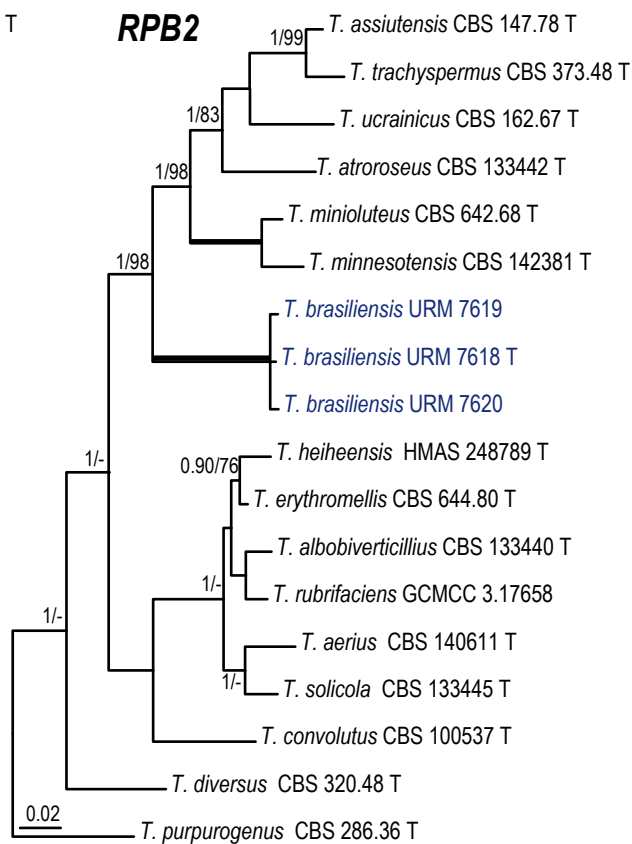

Supplement: Supplementary file 9 — Supplementary material 9 (PDF 249 kb) [file 10482_2018_1081_MOESM9_ESM.pdf]
